# Discovery of novel isoliquiritigenin analogue ISL-17 as a potential anti-gastric cancer agent

**DOI:** 10.1042/BSR20201199

**Published:** 2020-06-19

**Authors:** Fengchang Huang, Jin Wang, Yi Xu, Yunfei Zhang, Ning Xu, Liang Yin

**Affiliations:** 1 Department of Oncology, The First Affiliated Hospital of Kunming Medical University, Kunming 650032, Yunnan Province, China; 2 Department of Vascular Surgery, The First Affiliated Hospital of Kunming Medical University, Kunming 650032, Yunnan Province, China; 3 Department of Pharmacy, The First Affiliated Hospital of Wenzhou Medical University, Wenzhou 325000, Zhejiang, China

**Keywords:** Apoptosis, Cell cycle, Drug-likeness, Gastric cancer, Isoliquiritigenin analogues, Synthesis

## Abstract

Isoliquiritigenin (ISL), a natural product isolated from licorice root, exhibits anti-gastric cancer effects. However, applications of ISL are still limited in clinical practice due to its poor bioavailability. To discovery of more effective anti-gastric cancer agents based on ISL, aldol condensation reaction was applied to synthesize the ISL analogues. MTS assay was used to evaluate the inhibitory activities of ISL analogues against SGC-7901, BGC-823 and GES-1 cells *in vitro*. Cell cycle distribution, apoptosis and reactive oxygen species (ROS) generation were detected by flow cytometry. Western blot assay was used to analyze the expression levels of related proteins. The drug-likeness and pharmacokinetic properties were predicted with Osiris property explorer and PreADMET server. As a result, 18 new ISL analogues (**ISL-1** to **ISL-18**) were synthesized. Among these analogues, **ISL-17** showed the strongest inhibitory activities against SGC-7901 and BGC-823 cells, and could induce G2/M cell cycle arrest and apoptosis in these two cell lines. Treatment with **ISL-17** resulted in increased ROS production and elevated autophagy levels in SGC-7901 cells. The PI3K/AKT/mTOR signaling pathway was down-regulated after treatment with **ISL-17** in SGC-7901 cells. The results of drug-likeness and pharmacokinetic prediction indicated that all the ISL analogues complied with *Lipinski's rule of five* and *Veber rule* and had a favorable ADME character. Overall, our results attest that **ISL-17** holds promise as a candidate agent against gastric cancer.

## Introduction

Gastric cancer has a high morbidity and mortality worldwide and is one of the most common malignant tumors in the human digestive system [1]. Patients with gastric cancer generally have a low survival rate, which is partly caused by heterogeneity, a condition that is especially prominent among patients with advanced gastric cancer [2]. Current treatments for gastric cancer have several well-documented limitations, such as severe adverse effects of radiotherapy and chemotherapy, as well as resistance to certain anticancer drugs. In the clinic, surgical resection remains an important method for the treatment of gastric cancer [3]. Although new technologies such as gene therapy have been developed in recent years, the high cost makes it difficult to become widely applied as a viable treatment [4]. As a result, there remains an urgent need to discover new anti-gastric cancer drugs that can overcome the aforementioned problems.

**Figure 1:**
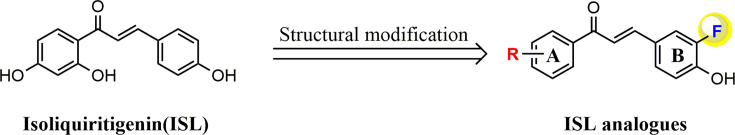
Design strategy of novel ISL analogues

Isoliquiritigenin (ISL, Figure 1) is a natural product that originates from the roots of licorice plants, including *Glycyrrhiza uralensis, Mongolian glycyrrhiza*, and so forth [5]. A number of previous studies have confirmed that ISL can function effectively as a versatile molecule. For example, ISL can be used as a food additive and has broad biological activities, such as antitumor [6], antimicrobial [7], antiviral [8], anti-inflammatory [9] and anti-oxidative activities [10], etc. In terms of its antitumor effects, ISL has been found to exhibit a therapeutic effect on multiple cancer types, including breast, cervical, liver, prostate, colon and gastric cancer [11–16], etc. The antitumor mechanisms of ISL can be mainly attributed to its role in affecting tumor formation, growth, proliferation and metastasis, etc. Although ISL seems like a powerful multifunctional molecule, its poor bioavailability [17] and water solubility [18] limit its further clinical application.

To find more potent anti-gastric cancer agents, we decided to optimize the structure of ISL. In the development of new drugs, fluorine is a chemical element that has been widely used due to its unique chemical properties. Among the fluorine-containing drugs approved by the FDA, fluorine exists in various structural forms such as ArF, AlkF and ArCF_3_, which were often used to improve the performance of a certain agent [19]. For this reason, a fluorine atom was introduced to the structure of ISL in this article. We designed and synthesized a series of 18 ISL analogues and evaluated their effects on human gastric cancer cell lines. First, we tested the inhibitory activity of these ISL analogues in two different gastric cancer cell lines (SGC-7901 and BGC-823 cells) and human gastric mucosal cells (GES-1 cells). Then, the underlying antitumor mechanisms of the most active compound in human gastric cancer cells were also evaluated, elucidating how it affects cell cycle distribution, apoptosis, autophagy and generation of reactive oxygen species (ROS), and exerts its effect on cell signaling pathways. Finally, we also performed some drug-likeness analysis on the ISL analogues.

## Materials and methods

### Chemicals and instruments

Isoliquiritigenin (ISL) was purchased from Energy Chemical (Shanghai, China), and dimethyl sulfoxide (DMSO) was procured from Sigma–Aldrich (St. Louis, CA). All commercial products were used without further purification. Eighteen ISL analogues were synthesized in our laboratory, with all their reactions monitored by thin layer chromatography (TLC) and purified by silica gel column chromatography. Melting points were performed in capillary tubes on SGW^®^ X-4 melting apparatus (Shanghai Shenguang Instrument and Meter Co., Ltd, China). The structures of all synthesized compounds were analyzed by high-resolution mass spectra (HRMS) and nuclear magnetic resonance (NMR) instrument. HRMS spectra of compounds were recorded on SHIMADZU LCMS-IT-TOF, which was equipped with an electrospray ion source (ESI) operating in positive ion mode. ^1^H NMR and ^13^C NMR spectra of compounds were tested on a 400 or 500 MHz apparatus (Bruker Corporation, Ettlingen, Germany) using tetramethylsilane (TMS) as an internal standard.

### General procedure for the preparation of ISL-1 to ISL-18

In a reaction flask, acetophenone or substituted acetophenone (1 mmol) and 3-fluoro-4-hydroxybenzaldehyde (1 mmol) was dissolved in ethanol (10 ml). Sixty percent KOH (0.6 ml) was added and the mixture stirred under reflux at 120°C for 3–5 h. TLC was used to detect reaction progress. When the reaction was completed, ethanol was distilled off under reduced pressure, extracted and purified by silica gel column chromatography to obtain the final desired products. Except **ISL-16** [20], the structures of all the other ISL analogues in this article were not reported.

Data on the characteristics of the ISL analogues (**ISL-1** to **ISL-18**) were presented as follows:

(*E*)-3-(3-fluoro-4-hydroxyphenyl)-1-phenylprop-2-en-1-one (**ISL-1**)
  Yellow powder. Yield: 70%. m.p.: 152–153°C. ^1^H NMR (500 MHz, DMSO-*d*_6_) δ (ppm): 10.54 (1H, s, 4′-OH), 8.14 (2H, d, *J = 7.5 Hz*, H-2, H-6), 7.86 (1H, dd, *J_1_ = 13 Hz, J_2_ = 2.0 Hz*, Ar-COCH = CH-Ar), 7.81 (1H, d, *J = 15.5 Hz*, H-6′), 7.68-7.63 (2H, m, Ar-COCH = CH-Ar, H-4), 7.58–7.55 (2H, m, H-3, H-5), 7.50 (1H, d, *J_1_ = 8.5 Hz, J_2_ = 1.5 Hz*, H-5′), 7.00 (1H, t, *J = 8.5 Hz*, H-2′). ^13^C NMR (125 MHz, DMSO-*d*_6_) δ (ppm): 188.8, 152.0, 147.5, 143.3, 137.7, 132.9, 128.7 × 3, 128.4 × 2, 127.0, 119.9, 117.7, 115.8. HRMS (ESI, *m/z*) calcd. for C_15_H_11_FO_2_ (M-H)^−^ 241.0670, found 241.0693.
(*E*)-3-(3-fluoro-4-hydroxyphenyl)-1-(o-tolyl)prop-2-en-1-one (**ISL-2**)
  Yellow powder. Yield: 77%. m.p.: 110–111°C. ^1^H NMR (400 MHz, DMSO-*d*_6_) δ (ppm): 10.54 (1H, s, 4′-OH), 7.71 (1H, dd, *J_1_ = 12.8 Hz, J_2_ = 2.0 Hz*, Ar-COCH = CH-Ar), 7.58 (1H, dd, *J_1_ = 8.4 Hz, J_2_ = 1.6 Hz*, H-6), 7.44–7.26 (5H, m, H-3, H-4, H-5, H-6′, Ar-COCH = CH-Ar), 7.24 (1H, d, *J = 16 Hz*, H-5′), 6.97 (1H, t, *J = 8.4 Hz*, H-2′), 2.53 (3H, s, 2-CH_3_). ^13^C NMR (100 MHz, DMSO-*d*_6_) δ (ppm): 194.8, 152.2, 147.6, 144.1, 138.9, 136.1, 131.1, 130.4, 128.1, 126.6, 126.1, 125.6, 124.3, 117.8, 115.9, 19.8. HRMS (ESI, *m/z*) calcd. for C_16_H_13_FO_2_ (M-H)^−^ 255.0827, found 255.0846.
(*E*)-3-(3-fluoro-4-hydroxyphenyl)-1-(2-fluorophenyl)prop-2-en-1-one (**ISL-3**)
  Yellow powder. Yield: 55%. m.p.: 105–106°C. ^1^H NMR (400 MHz, DMSO-*d*_6_) δ (ppm): 10.60 (1H, s, 4′-OH), 7.76–7.61 (3H, m, Ar-COCH = CH-Ar, Ar-COCH = CH-Ar, H-6), 7.53 (1H, d, *J = 16 Hz*, H-6′), 7.44 (1H, dd, *J_1_ = 8.4 Hz, J_2_ = 1.2 Hz*, H-3), 7.38–7.28 (3H, m, H-4, H-5, H-5′), 6.99 (1H, t, *J = 8.8 Hz*, H-2′). ^13^C NMR (100 MHz, DMSO-*d*_6_) δ (ppm): 188.8, 161.2, 152.2, 147.7, 144.1, 133.9, 133.8, 130.3, 127.2, 126.6, 124.7, 123.6, 117.8, 116.6, 115.9. HRMS (ESI, *m/z*) calcd. for C_15_H_10_F_2_O_2_ (M-H)^−^ 259.0576, found 259.0596.
(*E*)-1-(2-bromophenyl)-3-(3-fluoro-4-hydroxyphenyl)prop-2-en-1-one (**ISL-4**)
Yellowish powder. Yield: 66%. m.p.: 145–146°C. ^1^H NMR (400 MHz, DMSO-*d*_6_) δ (ppm): 10.62 (1H, s, 4′-OH), 7.73 (1H, d, *J = 8.0 Hz*, H-6), 7.69 (1H, dd, *J_1_ = 12.8 Hz, J_2_ = 2.0 Hz*, Ar-COCH = CH-Ar), 7.53–7.42 (3H, m, H-4, H-5, Ar-COCH = CH-Ar), 7.39 (1H, dd, *J_1_ = 8.4 Hz, J_2_ = 1.6 Hz*, H-3), 7.26 (1H, d, *J = 16 Hz*, H-6′), 7.09 (1H, d, *J = 16 Hz*, H-5′), 6.96 (1H, t, *J = 8.4 Hz*, H-2′). ^13^C NMR (100 MHz, DMSO-*d*_6_) δ (ppm): 193.9, 152.2, 149.8, 145.9, 140.8, 133.0, 131.6, 128.9, 127.7, 126.8, 124.1, 118.5, 117.8, 116.1, 115.9. HRMS (ESI, *m/z*) calcd. for C_15_H_10_BrFO_2_ (M-H)^−^ 318.9775, found 318.9800.
(*E*)-1-(3-aminophenyl)-3-(3-fluoro-4-hydroxyphenyl)prop-2-en-1-one (**ISL-5**)
  Yellow powder. Yield: 77%. m.p.: 207–208°C. ^1^H NMR (400 MHz, DMSO-*d*_6_) δ (ppm): 10.49 (1H, s, 4′-OH), 7.78 (1H, dd, *J_1_ = 12.8 Hz, J_2_ = 2.0 Hz*, Ar-COCH = CH-Ar), 7.65 (1H, d, *J = 15.6 Hz*, Ar-COCH = CH-Ar), 7.59 (1H, d, *J = 15.6 Hz*, H-6′), 7.46 (1H, d, *J = 8.4 Hz*, H-6), 7.32 (1H, d, *J = 7.6 Hz*, H-5), 7.24 (1H, s, H-2), 7.18 (1H, t, *J = 8.0 Hz*, H-5′), 6.99 (1H, t, *J = 8.8 Hz*, H-2′), 6.82 (1H, dd, *J_1_ = 8.0 Hz, J_2_ = 1.6 Hz*, H-4), 5.33 (2H, s, 3-NH_2_). ^13^C NMR (100 MHz, DMSO-*d*_6_) δ (ppm): 189.3, 152.3, 149.0, 147.2, 142.6, 138.5, 129.0, 126.5, 120.4, 118.4, 117.7, 116.3, 115.7, 115.5, 112.9. HRMS (ESI, *m/z*) calcd. for C_15_H_12_FNO_2_ (M-H)^−^ 256.0779, found 256.0797.
(*E*)-3-(3-fluoro-4-hydroxyphenyl)-1-(4-fluorophenyl)prop-2-en-1-one (**ISL-6**)
  Yellow powder. Yield: 55%. m.p.: 205–206°C. ^1^H NMR (400 MHz, DMSO-*d*_6_) δ (ppm): 10.54 (1H, s, 4′-OH), 8.26–8.22 (2H, m, H-2, H-6), 7.86 (1H, d, *J = 12.8 Hz*, Ar-COCH = CH-Ar), 7.71 (1H, d, *J = 15.2 Hz*, Ar-COCH = CH-Ar), 7.66 (1H, d, *J = 15.6 Hz*, H-6′), 7.50 (1H, d, *J = 8.4 Hz*, H-5′), 7.38 (2H, t, *J = 8.8 Hz*, H-3, H-5), 6.99 (1H, t, *J = 8.8 Hz*, H-2′). ^13^C NMR (100 MHz, DMSO-*d*_6_) δ (ppm): 187.3, 166.2, 152.3, 149.9, 147.5, 134.3, 131.3 × 2, 127.0, 126.5, 119.7, 117.7, 115.8 × 2, 115.7. HRMS (ESI, *m/z*) calcd. for C_15_H_10_F_2_O_2_ (M-H)^−^ 259.0576, found 259.0596.
(*E*)-1-(4-aminophenyl)-3-(3-fluoro-4-hydroxyphenyl)prop-2-en-1-one (**ISL-7**)
  Yellow powder. Yield: 50%. m.p.: 191–192°C. ^1^H NMR (400 MHz, DMSO-*d*_6_) δ (ppm): 10.38 (1H, s, 4′-OH), 7.91 (2H, d, *J = 8.4 Hz*, H-2, H-6), 7.78 (1H, d, *J = 12.4 Hz*, Ar-COCH = CH-Ar), 7.72 (1H, d, *J = 15.2 Hz*, Ar-COCH = CH-Ar), 7.52 (1H, d, *J = 15.6 Hz*, H-6′), 7.43 (1H, d, *J = 8.0 Hz*, H-5′), 6.97 (1H, t, *J = 8.8 Hz*, H-2′), 6.61 (2H, d, *J = 8.4 Hz*, H-3, H-5), 6.11 (2H, 4-NH_2_). ^13^C NMR (100 MHz, DMSO-*d*_6_) δ (ppm): 185.7, 153.7, 152.3, 149.9, 146.7, 130.9 × 2, 127.1, 126.2, 125.4, 120.4, 117.6, 115.4, 112.6 × 2. HRMS (ESI, *m/z*) calcd. for C_15_H_12_FNO_2_ (M-H)^−^ 256.0779, found 256.0799.
(*E*)-3-(3-fluoro-4-hydroxyphenyl)-1-(p-tolyl)prop-2-en-1-one (**ISL-8**)
Orange powder. Yield: 65%. m.p.: 149–150°C. ^1^H NMR (400 MHz, DMSO-*d*_6_) δ (ppm): 10.50 (1H, s, 4′-OH), 8.05 (2H, d, *J = 8.0 Hz*, H-2, H-6), 7.85 (1H, dd, *J_1_ = 12.8 Hz, J_2_ = 2.0 Hz*, Ar-COCH = CH-Ar), 7.79 (1H, d, *J = 15.6 Hz*, Ar-COCH = CH-Ar), 7.63 (1H, d, *J = 15.2 Hz*, H-6′), 7.49 (1H, dd, *J_1_ = 8.4 Hz, J_2_ = 1.6 Hz*, H-5′), 7.36 (2H, d, *J = 8.0 Hz*, H-3, H-5), 6.99 (1H, t, *J = 8.8 Hz*, H-2′), 2.39 (3H, s, 4-CH_3_). ^13^C NMR (100 MHz, DMSO-*d*_6_) δ (ppm): 188.2, 152.3, 149.9, 147.5, 143.3, 135.1, 129.2 × 2, 128.5 × 2, 126.8, 126.7, 126.6, 119.9, 117.7, 21.1. HRMS (ESI, *m/z*) calcd. for C_16_H_13_FO_2_ (M-H)^−^ 255.0827, found 255.0848.
(*E*)-3-(3-fluoro-4-hydroxyphenyl)-1-(4-(trifluoromethyl)phenyl)prop-2-en-1-one (**ISL-9**)
  Yellow powder. Yield: 53%. m.p.: 131–132°C. ^1^H NMR (400 MHz, DMSO-*d*_6_) δ (ppm): 10.60 (1H, s, 4′-OH), 8.30 (2H, d, *J = 8.0 Hz*, H-2, H-6), 7.91 (2H, d, *J = 8.4 Hz*, H-3, H-5), 7.87 (1H, dd, *J_1_ = 12.8 Hz, J_2_ = 2.0 Hz*, Ar-COCH = CH-Ar), 7.81 (1H, d, *J = 15.6 Hz*, Ar-COCH = CH-Ar), 7.70 (1H, d, *J = 15.6 Hz*, H-6′), 7.52 (1H, d, *J_1_ = 8.4 Hz, J_2_ = 1.6 Hz*, H-5′), 7.00 (1H, t, *J = 8.8 Hz*, H-2′). ^13^C NMR (100 MHz, DMSO-*d*_6_) δ (ppm): 188.2, 152.3, 149.9, 147.8, 144.5, 140.9, 129.1 × 2, 127.2, 126.4, 125.7, 125.6 × 2, 119.7, 117.7, 115.8. HRMS (ESI, *m/z*) calcd. for C_16_H_10_F_4_O_2_ (M-H)^−^ 309.0544, found 309.0566.
(*E*)-4-(3-(3-fluoro-4-hydroxyphenyl)acryloyl)benzonitrile (**ISL-10**)
  Yellow powder. Yield: 63%. m.p.: 198–199°C. ^1^H NMR (400 MHz, DMSO-*d*_6_) δ (ppm): 10.61 (1H, s, 4′-OH), 8.27 (2H, d, *J = 8.4 Hz*, H-3, H-5), 8.04 (2H, d, *J = 8.8 Hz*, H-2, H-6), 7.88 (1H, dd, *J_1_ = 12.8 Hz, J_2_ = 2.0 Hz*, Ar-COCH = CH-Ar), 7.80 (1H, d, *J = 15.6 Hz*, Ar-COCH = CH-Ar), 7.70 (1H, d, *J = 15.6 Hz*, H-6′), 7.52 (1H, d, *J_1_ = 8.4 Hz, J_2_ = 1.6 Hz*, H-5′), 7.00 (1H, t, *J = 8.8 Hz*, H-2′′). ^13^C NMR (100 MHz, DMSO-*d*_6_) δ (ppm): 188.0, 152.3, 149.9, 147.9, 144.7, 132.7 × 2, 129.8 × 2, 128.9, 127.3, 119.5, 118.2, 117.7, 116.0, 114.8. HRMS (ESI, *m/z*) calcd. for C_16_H_10_FNO_2_ (M-H)^−^ 266.0623, found 266.0644.
(*E*)-3-(3-fluoro-4-hydroxyphenyl)-1-(4-methoxyphenyl)prop-2-en-1-one (**ISL-11**)
  Yellow powder. Yield: 52%. m.p.: 123–124°C. ^1^H NMR (400 MHz, DMSO-*d*_6_) δ (ppm): 10.48 (1H, s, 4′-OH), 8.15 (2H, d, *J = 8.8 Hz*, H-2, H-6), 7.86-7.78 (2H, m, Ar-COCH = CH-Ar), 7.61 (1H, d, *J = 15.6 Hz*, H-6′), 7.48 (1H, dd, *J_1_ = 8.0 Hz, J_2_ = 1.6 Hz*, H-5′), 7.07 (2H, d, *J = 8.8 Hz*, H-3, H-5), 6.99 (1H, t, *J = 8.8 Hz*, H-2′), 3.86 (3H, s, 4-OCH_3_). ^13^C NMR (100 MHz, DMSO-*d*_6_) δ (ppm): 187.1, 163.0, 152.3, 149.9, 147.3, 130.7 × 2, 130.5, 126.7, 119.9, 117.7, 115.7, 115.5, 113.9 × 2, 55.5. HRMS (ESI, *m/z*) calcd. for C_16_H_13_FO_3_ (M-H)^−^ 271.0776, found 271.0798.
(*E*)-1-(4-(dimethylamino)phenyl)-3-(3-fluoro-4-hydroxyphenyl)prop-2-en-1-one (**ISL-12**)
  Yellow powder. Yield: 88%. m.p.: 214–215°C. ^1^H NMR (400 MHz, DMSO-*d*_6_) δ (ppm): 10.40 (1H, s, 4′-OH), 8.03 (2H, d, *J = 9.2 Hz*, H-2, H-6), 7.82–7.74 (2H, m, Ar-COCH = CH-Ar, H-6′), 7.54 (1H, d, *J = 15.6 Hz*, Ar-COCH = CH-Ar), 7.45 (1H, d, *J = 8.0 Hz*, H-5′), 6.98 (1H, t, *J = 8.4 Hz*, H-2′), 6.75 (2H, d, *J = 8.8 Hz*, H-3, H-5), 3.04 (6H, s, 4-N(CH_3_)_2_). ^13^C NMR (100 MHz, DMSO-*d*_6_) δ (ppm): 185.9, 153.2, 152.3, 147.0, 140.9, 130.5 × 2, 126.3, 125.2, 120.3, 117.7, 115.5, 110.7 × 2, 110.5, 39.5 × 2. HRMS (ESI, *m/z*) calcd. for C_17_H_16_FNO_2_ (M-H)^−^ 284.1092, found 284.1114.
(*E*)-1-(2,3-dichlorophenyl)-3-(3-fluoro-4-hydroxyphenyl)prop-2-en-1-one (**ISL-13**).
Yellow powder. Yield: 72%. m.p.: 169–170°C. ^1^H NMR (400 MHz, DMSO-*d*_6_) δ (ppm): 10.64 (1H, s, 4′-OH), 7.78 (1H, dd, *J_1_ = 6.8 Hz, J_2_ = 2.8 Hz*, H-4), 7.70 (1H, dd, *J_1_ = 12.4 Hz, J_2_ = 1.6 Hz*, Ar-COCH = CH-Ar), 7.51–7.46 (2H, m, H-5, Ar-COCH = CH-Ar), 7.42 (1H, dd, *J_1_ = 8.4 Hz, J_2_ = 1.6 Hz*, H-6), 7.30 (1H, d, *J = 16 Hz*, H-6′), 7.09 (1H, d, *J = 16 Hz*, H-5′), 6.96 (1H, t, *J = 8.4 Hz*, H-2′). ^13^C NMR (100 MHz, DMSO-*d*_6_) δ (ppm): 192.5, 152.2, 148.2, 146.7, 141.1, 132.3, 131.7, 128.6, 127.7, 127.3, 127.0, 124.1, 117.8, 116.3, 116.1. HRMS (ESI, *m/z*) calcd. for C_15_H_9_Cl_2_FO_2_ (M-H)^−^ 308.9891, found 308.9920
(*E*)-1-(4-fluoro-2-hydroxyphenyl)-3-(3-fluoro-4-hydroxyphenyl)prop-2-en-1-one (**ISL-14**)
  Yellow powder. Yield: 75%. m.p.: 215–216°C. ^1^H NMR (400 MHz, DMSO-*d*_6_) δ (ppm): 13.22 (1H, s, 2-OH), 10.65 (1H, s, 4′-OH), 8.43–8.39 (1H, m, Ar-COCH = CH-Ar), 7.92–7.85 (2H, m, H-6, Ar-COCH = CH-Ar), 7.77 (1H, d, *J = 15.2 Hz*, H-6′), 7.53 (1H, d, *J = 8.0 Hz*, H-5), 7.01 (1H, t, *J = 8.8 Hz*, H-2′), 6.89–6.82 (2H, m, H-3, H-5′). ^13^C NMR (100 MHz, DMSO-*d*_6_) δ (ppm): 192.4, 167.7, 164.6, 152.3, 149.9, 144.7, 133.7, 127.6, 126.2, 119.1, 117.7, 117.6, 116.0, 107.0, 104.3. HRMS (ESI, *m/z*) calcd. for C_15_H_10_F_2_O_3_ (M-H)^−^ 275.0525, found 275.0549.
(*E*)-1-(4-amino-3,5-dichlorophenyl)-3-(3-fluoro-4-hydroxyphenyl)prop-2-en-1-one (**ISL-15**)
  Yellow powder. Yield: 49%. m.p.: 230–231°C. ^1^H NMR (400 MHz, CD_3_OD) δ (ppm): 8.01 (2H, s, H-2, H-6), 7.67 (1H, d, *J = 16 Hz*, Ar-COCH = CH-Ar), 7.59–7.56 (2H, m, H-6′, Ar-COCH = CH-Ar), 7.39 (1H, d, *J = 8.4 Hz*, H-5′), 6.95 (1H, t, *J = 8.8 Hz*, H-2′). ^13^C NMR (100 MHz, CD_3_OD) δ (ppm): 188.0, 149.0, 145.0, 138.7, 137.5, 130.0 × 2, 128.6, 128.3 × 2, 127.5, 125.8, 120.1, 119.5, 116.7. HRMS (ESI, *m/z*) calcd. for C_15_H_10_Cl_2_FNO_2_ (M-H)^−^ 324.0000, found 324.0020.
(*E*)-3-(3-fluoro-4-hydroxyphenyl)-1-(2,3,4-trimethoxyphenyl)prop-2-en-1-one (**ISL-16**)
  Yellow powder. Yield: 51%. m.p.: 81–82°C. ^1^H NMR (400 MHz, CDCl_3_) δ (ppm): 7.58 (1H, d, *J = 16 Hz*, Ar-COCH = CH-Ar), 7.48 (1H, d, *J = 8.8 Hz*, H-6), 7.38–7.34 (2H, m, H-6′, Ar-COCH = CH-Ar), 7.29 (1H, d, *J = 8.4 Hz*, H-5), 7.01 (1H, t, *J = 8.8 Hz*, H-2′), 6.75 (1H, d, *J = 8.8 Hz*, H-5′), 3.92 (3H, s, 2-OCH_3_), 3.91 (3H, s, 4-OCH_3_), 3.91 (3H, s, 3-OCH_3_). ^13^C NMR (100 MHz, CDCl_3_) δ (ppm): 190.6, 157.1, 153.7, 152.3, 145.7, 142.1, 141.8, 126.7, 125.9, 125.8, 125.5, 117.6, 114.9, 114.7, 107.3, 62.1, 61.1, 56.1. HRMS (ESI, *m/z*) calcd. for C_18_H_17_FO_5_ (M-H)^−^ 331.0987, found 331.1014.
(*E*)-3-(3-fluoro-4-hydroxyphenyl)-1-(3,4,5-trimethoxyphenyl)prop-2-en-1-one (**ISL-17**)
  Yellow powder. Yield: 70%. m.p.: 162–163°C. ^1^H NMR (500 MHz, DMSO-*d*_6_) δ (ppm): 10.53 (1H, s, 4′-OH), 7.88 (1H, dd, *J_1_ = 12.5 Hz, J_2_ = 2.0 Hz*, Ar-COCH = CH-Ar), 7.79 (1H, d, *J = 15.5 Hz*, Ar-COCH = CH-Ar), 7.66 (1H, d, *J = 15.5 Hz*, H-6′), 7.52 (1H, d, *J_1_ = 8.5 Hz, J_2_ = 1.5 Hz*, H-5′), 7.41 (2H, s, H-2, H-6), 7.00 (1H, t, *J = 8.5 Hz*, H-2′), 3.90 (6H, s, 3,5-OCH_3_), 3.76 (3H, s, 4-OCH_3_). ^13^C NMR (125 MHz, DMSO-*d*_6_) δ (ppm): 187.5, 152.8 × 3, 147.4, 143.2, 141.8, 133.1, 127.1, 126.6, 119.7, 117.7, 115.9, 106.0 × 2, 60.1, 56.1 × 2. HRMS (ESI, *m/z*) calcd. for C_18_H_17_FO_5_ (M-H)^−^ 331.0987, found 331.1014.
(*E*)-3-(3-fluoro-4-hydroxyphenyl)-1-(naphthalen-2-yl)prop-2-en-1-one (**ISL-18**)
  Yellow powder. Yield: 63%. m.p.: 165–166°C. ^1^H NMR (400 MHz, DMSO-*d*_6_) δ (ppm): 10.56 (1H, s, 4′-OH), 8.92 (1H, s, H-1), 8.16–8.12 (2H, m, H-4, H-8), 8.06–7.98 (3H, m, Ar-COCH = CH-Ar, H-3, H-5), 7.91 (1H, dd, *J_1_ = 12.4 Hz, J_2_ = 2.0 Hz*, Ar-COCH = CH-Ar), 7.73 (1H, d, *J = 16 Hz*, H-6′), 7.69–7.63 (2H, m, H-5, H-6), 7.55 (1H, d, *J_1_ = 8.4 Hz, J_2_ = 1.6 Hz*, H-5′), 7.02 (1H, t, *J = 8.8 Hz*, H-2′). ^13^C NMR (100 MHz, DMSO-*d*_6_) δ (ppm): 188.5, 152.3, 149.9, 147.6, 143.2, 135.0, 134.9, 132.3, 130.2, 129.5, 128.5, 128.3, 127.6, 127.0, 126.9, 124.1, 120.0, 117.7, 115.8. HRMS (ESI, *m/z*) calcd. for C_19_H_13_FO_2_ (M-H)^−^ 291.0827, found 291.0851.

### Cell lines and cell culture

Human gastric cancer cell lines SGC-7901 and BGC-823 were purchased from Chinese Academy of Sciences, a typical cell library culture preservation committee (Shanghai, China) and human gastric mucosal cell line GES-1 were purchased from Shanghai Honsun Biological Technology Co., Ltd. (Shanghai, China). RPMI 1640 medium and phosphate buffered saline (PBS) were purchased from Zhejiang Senrui Biotechnology Co., Ltd (Zhejiang, China). 3-(4,5-Dimethylthiazol-2-yl)-5-(3-carboxymethoxyphenyl)-2-(4-sulfophenyl)-2H-tetrazolium (MTS) was purchased from Promega (Madison, WI, U.S.A.). Cells were cultivated in RPMI 1640 medium containing 10% FBS (Gibco, Eggenstein, Germany) and incubated at 37°C in an atmosphere with 5% CO_2_.

### MTS assay

SGC-7901, BGC-823 and GES-1 cells were cultivated in RPMI 1640 medium containing 10% FBS. After being grown in the logarithmic phase, the cells were digested and counted. Five thousand cells per well were seeded in 96-well plates and incubated overnight. Then, the cells were treated with DMSO (negative control), ISL (positive control) and **ISL-1** to **ISL-18** at different concentrations for 48 h at 37°C. MTS solution was added to each well and incubated at 37°C for another 30 min. The absorbance was measured by a microplate reader (Molecular Devices, U.S.A.) at 490 nm. Stock solution (20 mM) of compounds was prepared with DMSO and freshly diluted with cell culture medium to different concentrations before use. For cell viability measurement, 100 μl of medium containing 20 μM of the tested compound (0.1 μl of stock solution) or DMSO (0.1 μl) was added to each well, and the diluting method of tested compound was similar for IC_50_ values measurement as well as other related cell experiments.

### Cell cycle assay

Cell cycle distributions were measured using a cell cycle detection kit (Beyotime, China). SGC-7901 and BGC-823 cells were seeded in six-well plates and incubated in a humidified 5% CO_2_ incubator at 37°C for 24 h. Afterwards, they were subjected to treatment with or without **ISL-17** (20 or 40 μM) and ISL (40 μM) for an additional 24 h. Seventy percent ice ethanol was used to fix the collected cells. After being fixed overnight, the cells were resuspended in PBS containing RNase A and PI (1:4, v/v) for 30 min. FACSCalibur flow cytometer (Bectone Dickinson, San Jose, CA, U.S.A.) was used to analyze DNA contents of the cells.

### Cell apoptosis assay

SGC-7901 and BGC-823 cells were seeded in six-well plates at a density of 5 × 10^5^ cells. When cells reach the logarithmic growth phase, they were treated with or without **ISL-17** (20 or 40 μM) and ISL (40 μM) for another 24 h. Then the cells were washed twice with ice-cold PBS and subjected to apoptosis analysis with Annexin V-FITC and PI (Beyotime, China) in binding buffer for 30 min. Cell apoptosis was analyzed by FACSCalibur flow cytometer.

### Intracellular ROS determined by flow cytometry

SGC-7901 cells were cultured in six-well plates for 24 h with 4 × 10^5^ cells per well before being treated with **ISL-17** (20 or 40 μM) for given time in the absence or presence of *N*-acetyl cysteine (NAC, 5 mM). Serum-free medium was mixed with DCFH-DA (10 μM) (Beyotime, China), and then incubated with cells for 30 min in a dark room at 37°C. Afterwards, the cells were collected and analyzed for ROS production by FACSCalibur flow cytometer.

### Western blot assay

Cells were plated in six-well plates. After treatment with **ISL-17** (20 or 40 μM) and ISL (40 μM) for the indicated time, the cells were scraped from the plate. The collected protein samples were then electrophoresed on 10% SDS/PAGE gels, and electroblotted onto a PVDF membrane. At room temperature, the membranes were blocked with 5% nonfat milk for 2 h. Primary antibodies were incubated with membranes at 4°C overnight. Then, the membranes were washed with TBST for three times and incubated with secondary antibody at room temperature for 1 h. The immune-reactive complexes were analyzed with ECL kit (Bio-Rad, Hercules, CA, U.S.A.). The anti-Bcl-2 (sc-7382, 1:1000) and anti-Bax antibody (sc-20067, 1:1000) was purchased from Santa Cruz Biotechnology (Santa Cruz, CA, U.S.A.). Antibodies including anti-Cyclin B1 (#4135, 1:2000), anti-Cdc2 (#9116, 1:1000), anti-Cleaved PARP (#5625, 1:1000), anti-p62 (#8025, 1:1000), anti-LC3B (#3868, 1:1000), anti-Beclin 1 (#3495, 1:1000), anti-AKT (#4691, 1:1000), anti-phospho-AKT (#4060, 1:2000), anti-mTOR (#2983, 1:1000), anti-phospho-mTOR (#5536, 1:1000), anti-rabbit IgG-HRP (#7074, 1:1000) and anti-mouse IgG-HRP (#7076, 1:1000) were purchased from Cell Signaling Technology (Danvers, MA, U.S.A.).

### Drug-likeness and ADMET prediction

The drug-likeness and pharmacokinetic properties of all the synthesized ISL analogues were predicted using related available online website, including Osiris property explorer [21] and PreADMET server [22].

### Statistical analysis

All experiments were replicated for a minimum of three times. Data analyses were performed using GraphPad Prism Software 6 (GraphPad Inc., San Diego, U.S.A.). The results were presented as means±SEMs. One-way and two-way analysis of variance (ANOVA) was conducted to compare differences between the groups, with a statistically significant result obtained at *P* < 0.05.

## Results

### Design and synthesis of ISL analogues

**Scheme 1:**
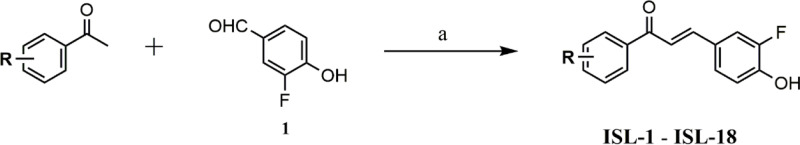
The general synthesis route of ISL analogues (**ISL-1** to **ISL-18**) Reagents and conditions – a: ethanol, 60% KOH, 120°C, reflux, 3–5 h.

Structural modification of ISL was carried out to find more potent and effective anti-gastric cancer agents. ISL belongs to the chalcone series. Considering that the α,β-unsaturated ketone is an important part of its biological activity [23], we keep this part, allowing the modification to take place on the aromatic rings on both sides (Figure 1). On the B ring of ISL, we introduced a fluorine atom adjacent to the hydroxyl group and then substituted it with different substituents on the A ring (Figure 1), including electron withdrawing group and electron donating group. Altogether, 18 ISL analogues (**ISL-1** to **ISL-18**) were designed (Table 1). Aldol condensation reaction was used to synthesize these ISL analogues with high yields. In brief, by dissolving 3-fluoro-4-hydroxybenzaldehyde (**1**) and different substituted acetophenones in ethanol, the reaction could be achieved under alkaline (60% KOH) and reflux conditions (Scheme 1). All the ISL analogues were characterized by HRMS, ^1^H NMR and ^13^C NMR. (Spectra of all compounds is displayed in ***Supplementary Information*** section.) The detailed characteristics of all products, including yield, color, melting points (m.p.), HRMS, ^1^H NMR and ^13^C NMR spectra of compounds were shown in *Chemicals and instruments* section.

**Table 1:** Structures of ISL analogues

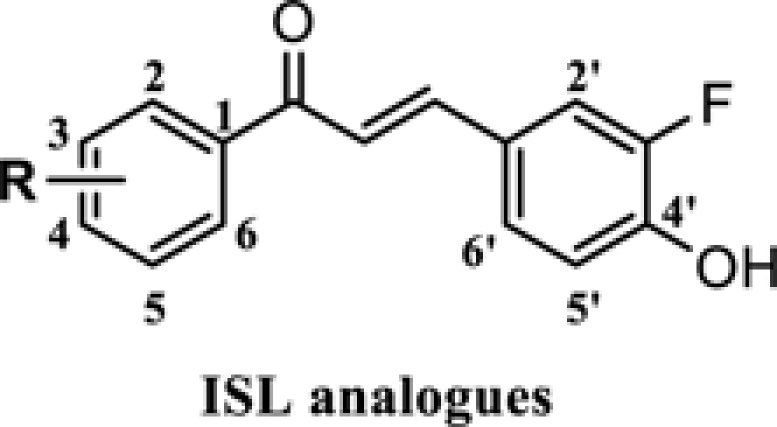			
Comp.	R	Comp.	R
**ISL-1**	-H	**ISL-10**	4-CN
**ISL-2**	2-CH_3_	**ISL-11**	4-OCH_3_
**ISL-3**	2-F	**ISL-12**	4-N(CH_3_)_2_
**ISL-4**	2-Br	**ISL-13**	2,3-Cl
**ISL-5**	3-NH_2_	**ISL-14**	2-OH, 4-F
**ISL-6**	4-F	**ISL-15**	3,5-Cl, 4-NH_2_
**ISL-7**	4-NH_2_	**ISL-16**	2,3,4-OCH_3_
**ISL-8**	4-CH_3_	**ISL-17**	3,4,5-OCH_3_
**ISL-9**	4-CF_3_	**ISL-18**	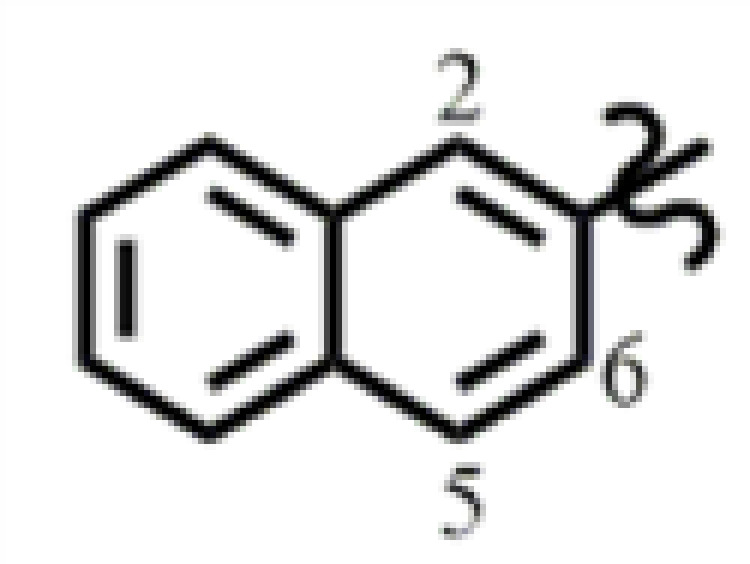

### In vitro screening of ISL analogues against human gastric cancer cells and normal gastric cells

**Figure 2:**
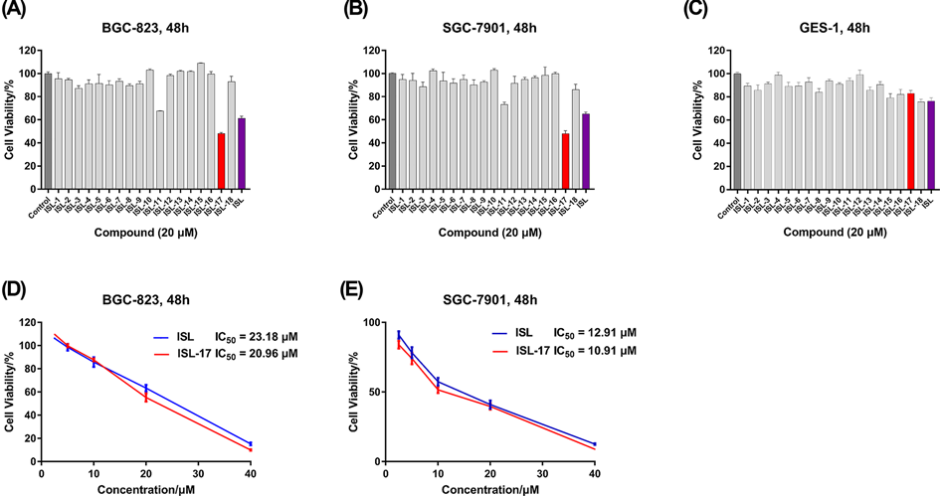
The growth inhibition rate of compounds against human gastric cancer cells and human gastric mucosal cells (**A–C**) BGC-823, SGC-7901 and GES-1 cells were treated with ISL analogues (**ISL-1** to **ISL-18**) or **ISL** at 20 μM for 48 h and then cell viability was determined by MTS assay. (**D,E**) BGC-823 and SGC-7901 cells were treated with **ISL-17** or **ISL** by concentration gradient (2.5, 5, 10, 20 and 40 μM) for 48 h, and then cell viability was determined by MTS assay.

The effect of ISL analogues on the growth of SGC-7901, BGC-823 and GES-1 cells was evaluated by MTS assay. As shown in Figure 2A,B, among all ISL analogues, only **ISL-17** could reach a 50% inhibition rate at a concentration of 20 μM when acting on BGC-823 and SGC-7901 cells, and is superior to ISL. For GES-1 cells, all the ISL analogues (20 μM) exhibited only a negligible effect on cell growth (Figure 2C) and cell viability exceeded 70%, while ISL had the strongest suppression effect in comparison. Furthermore, we determined the IC_50_ values of **ISL-17** against BGC-823 and SGC-7901 cells. The results showed that (Figure 2D,E) ISL-17 has a better inhibitory activity in both cell lines compared with ISL. The IC_50_ values of **ISL-17** against on BGC-823 and SGC-7901 cells were 20.96 and 10.91 μM, respectively, while the values of ISL were 23.18 and 12.91 μM, respectively. Based on the above results, we decided to take **ISL-17** as a representative compound in the next anticancer mechanistic study.

### ISL-17 induced G2/M cell cycle arrest in human gastric cancer cells

**Figure 3:**
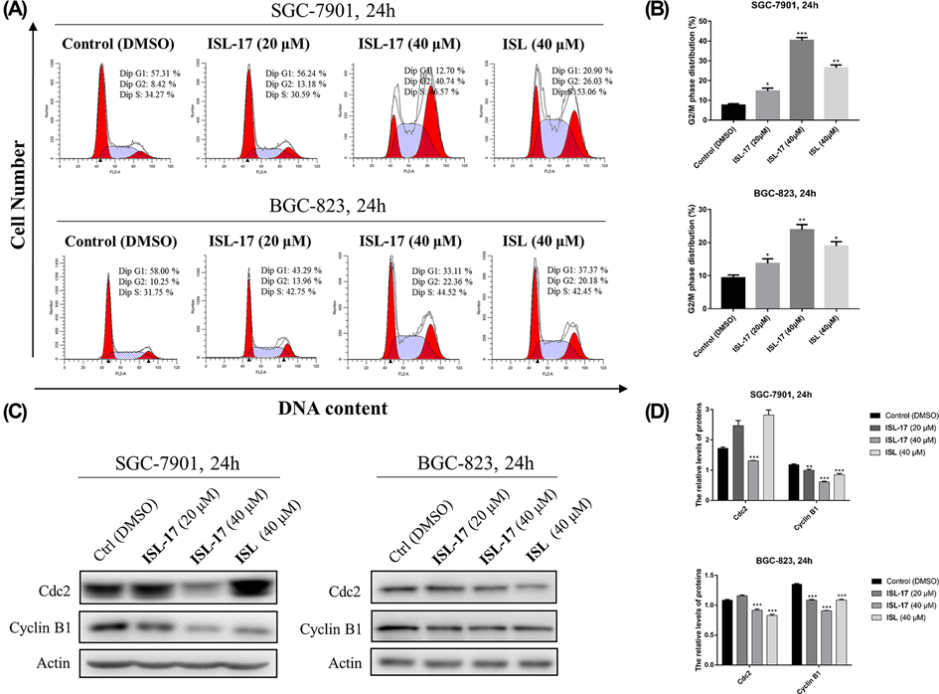
**ISL-17** induced cell cycle arrest in human gastric cancer cells (**A,B**) SGC-7901 and BGC-823 cells were treated with **ISL-17** (20 or 40 μM) or **ISL** (40 μM) for 24 h, and flow cytometer were used to analyze the cell cycle distribution. (**C,D**) Expression levels of G2/M cell cycle proteins Cdc2 and Cyclin B1 were determined by western blot after treatment with **ISL-17** (20 or 40 μM) or **ISL** (40 μM) for 24 h; actin was used as the internal control. Error bar original from three independent experiments (*P<0.05, **P<0.01, ***P<0.001 vs. control).

Previous studies have found that ISL can affect the cell cycle distribution in hepatocellular carcinoma cells [14] and prostate cancer cells [16], etc. However, research that has focused on human gastric cancer cells is few and far between. So, flow cytometry analysis was used to evaluate the effect of ISL and **ISL-17** on the cell cycle distributions of SGC-7901 and BGC-823 cells. As shown in Figure 3A,B, treatment with ISL and **ISL-17** arrested the cell cycle at G2/M phase. After treatment with **ISL-17** from 0 to 40 μM, the percentage of SGC-7901 cells in the G2/M phase increased from 8.42% to 40.74%, and from 10.25% to 22.36% in BGC-823 cells. Meanwhile, after incubation with ISL at a concentration of 40 μM, the percentage of SGC-7901 cells and BGC-823 cells in the G2/M phase was 26.03% and 20.18%, respectively.

Cdc2 kinase plays an important role in mitosis of eukaryotic cells, a process regulated by certain steps, including the binding of Cyclin B1 [24]. We further examined the expression levels of cell cycle-related proteins by western blot assay. The results showed that (Figure 3C,D), at a high concentration (40 μM) of **ISL-17**, the expression levels of Cdc2 and Cyclin B1 decreased in both SGC-7901 and BGC-823 cells. These results suggest that **ISL-17** could induce G2/M cell cycle arrest in SGC-7901 and BGC-823 cells.

### ISL-17 induced apoptosis in human gastric cancer cells

**Figure 4:**
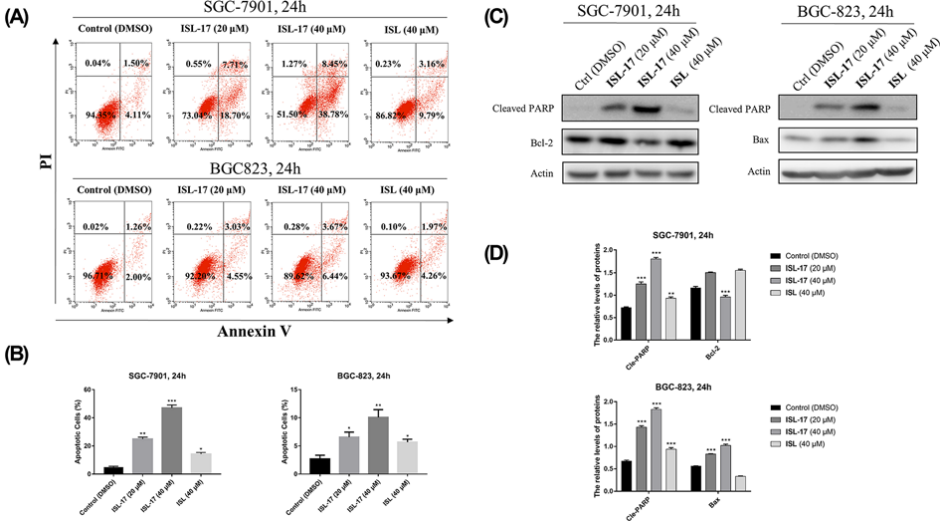
**ISL-17** induced apoptosis in human gastric cancer cells (**A**) Cell apoptosis was detected by flow cytometry. SGC-7901 and BGC-823 cells were treated with **ISL-17** or **ISL** for 24 h. Then apoptosis was detected. (**B**) Statistics of cell apoptosis. (**C,D**) The expression levels of apoptosis-related proteins analysis. Cells were treated with **ISL-17** or **ISL** for 24 h and the expression levels of apoptosis-related proteins were detected by western blot. Actin was used as the internal control. Error bar original from three independent experiments (*P<0.05, **P<0.01, ***P<0.001 vs. control).

Apoptosis belongs to programmed cell death and is one of the anticancer mechanisms [25]. In order to clarify whether **ISL-17** could induce apoptosis in human gastric cancer cells, SGC-7901 and BGC-823 cells were treated with **ISL-17** (20 or 40 μM) and ISL (40 μM) for 24 h. As illustrated in Figure 4A,B, the proportion of apoptosis cells increased (5.61–47.23%) in a dose-dependent manner after treatment with **ISL-17** from 0 to 40 μM, while the value was 12.95% for ISL at 40 μM. Furthermore, western blot assay was used to analyze the expression level of apoptosis-related proteins, such as Cleaved-PARP, Bcl-2 and Bax on SGC-7901 and BGC-823 cells. As shown in Figure 4C,D, the expression levels of Cleaved-PARP and Bax increased, while the expression level of Bcl-2 decreased after treatment with **ISL-17** (0–40 μM) and ISL (40 μM). These findings suggest that **ISL-17** could induce apoptosis in both SGC-7901 and BGC-823 cells.

### ISL-17 regulates ROS production in human gastric cancer cells

**Figure 5:**
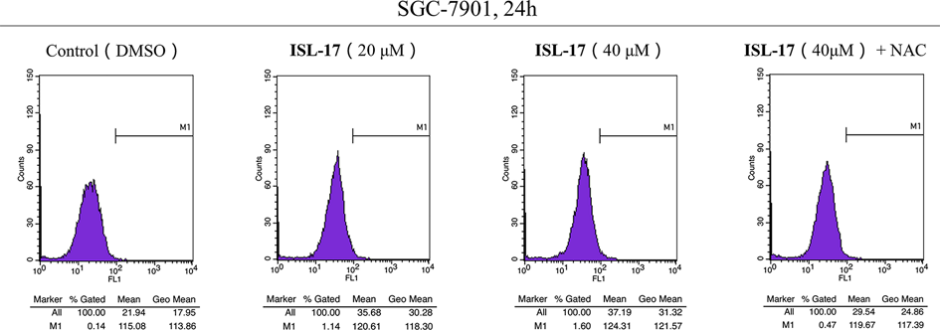
**ISL-17** induced ROS generation in human gastric cancer cells SGC-7901 cells were treated with **ISL-17** (20 or 40 μM) or **ISL-17** (40 μM) **+** NAC (5 mM) for 24 h, and then the generation of ROS was measured by flow cytometry.

The increase in ROS levels is one of the reasons for cancer cell growth [26], and further increasing the level of ROS has been shown to be an effective anticancer strategy [27]. Thus, DCFH-DA probe was used to detect the intracellular ROS levels after treatment with **ISL-17** in human gastric cancer cell lines. As illustrated in Figure 5, **ISL-17** induced intracellular ROS generation in a dose-dependent manner in SGC-7901 cells (mean value from 21.94 to 37.19), while the increased ROS was inhibited by pre-incubation with NAC (mean value was 29.54).

### ISL-17 induced autophagy in SGC-7901 cells

**Figure 6:**
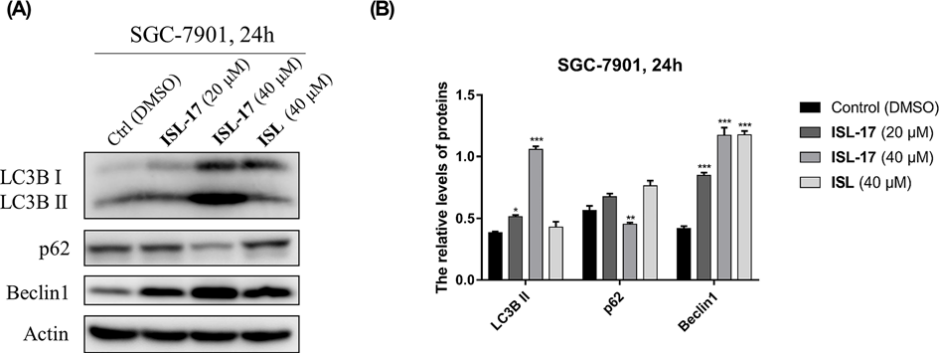
**ISL-17** induced autophagy in human gastric cancer cells (**A**) The expression levels of LC3B II, p62 and Beclin 1 were analyzed by western blot assay. (**B**) Statistics of the expression levels of LC3B II, p62 and Beclin 1 following **ISL-17** or **ISL** treatment. Actin was used as the internal control. Error bar original from three independent experiments (*P<0.05, **P<0.01, ***P<0.001 vs. control).

Autophagy is a vital intracellular homeostatic process and plays an important role in cancer progression, including gastric cancer [28]. To determine whether **ISL-17** could influence autophagy in human gastric cancer cells, western blot assay was used to detect the expression of autophagy-related proteins in SGC-7901 cells. As indicated in Figure 6A,B, the expression levels of LC3B II and Beclin 1 increased, whereas the expression level of p62 decreased after treatment with **ISL-17** from 0 to 40 μM. The results show that **ISL-17** may promote autophagy in SGC-7901 cells.

### ISL-17 down-regulates the PI3K/AKT/mTOR signaling pathway in SGC-7901 cells

**Figure 7:**
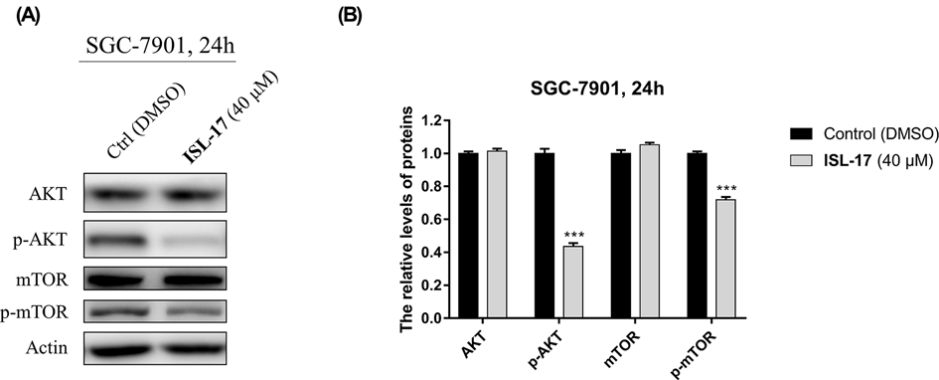
**ISL-17** down-regulates PI3K/AKT/mTOR signal pathway in SGC-7901 cells (**A**) The expression levels of AKT, p-AKT, mTOR and p-mTOR were analyzed by western blot assay after **ISL-17** treatment for 24 h in SGC-7901 cells. (**B**) Statistics of the expression levels of AKT, p-AKT, mTOR and p-mTOR. Actin was used as the internal control. Error bar original from three independent experiments (***P<0.001 vs. control).

PI3K/Akt/mTOR pathway is involved in the initiation and progression of gastric cancer [29]. The effect of **ISL-17** on this pathway in human gastric cancer cells was analyzed by western blot. As shown in Figure 7A,B, the expression levels of p-AKT and p-mTOR decreased, while their precursors AKT and m-TOR remained virtually unchanged after treatment with **ISL-17** (40 μM) for 24 h in SGC-7901 cells. These results indicate that **ISL-17** may exert its anticancer effect by interfering with PI3K/AKT/mTOR pathway in SGC-7901 cells.

### Physicochemical and pharmacokinetic properties of ISL analogues

In order to analyze the drug-likeness properties of ISL analogues, *Lipinski's rule of five* and *Veber rules* were used for evaluation. According to *Lipinski's rule of five* [30], small molecule drugs with good oral bioavailability should meet a molecular weight (MW) of less than 500 Da, a lipid-water partition coefficient (log *P*) of no more than 5, a hydrogen bond acceptor (HBA) of no more than 10, and a hydrogen bond donor (HBD) of no more than 5. *Veber rules* [31] generally state that small molecule drugs with good oral bioavailability should meet rotatable chemical bonds of no more than 10 and have a polar surface area (PSA) of no more than 140 Å^2^. The results in Table 2 indicate that all the ISL analogues obey the *Lipinski's rule of five* and *Veber rules* and demonstrate good properties.

**Table 2:** Molecular properties of ISL-1 to ISL-18 and ISL

Comp.	MW (g/mol)	log *P*	HBA	HBD	PSA (Å^2^)	Rule of five
**ISL**	256.2	2.26	4	3	77.76	Suitable
**ISL-1**	242.2	3.05	2	1	37.3	Suitable
**ISL-2**	256.2	3.40	2	1	37.3	Suitable
**ISL-3**	260.2	3.15	2	1	37.3	Suitable
**ISL-4**	321.1	3.78	2	1	37.3	Suitable
**ISL-5**	257.2	2.38	3	2	63.62	Suitable
**ISL-6**	260.2	3.15	2	1	37.3	Suitable
**ISL-7**	257.2	2.38	3	2	63.62	Suitable
**ISL-8**	256.2	3.40	2	1	37.3	Suitable
**ISL-9**	310.2	3.90	2	1	37.3	Suitable
**ISL-10**	267.2	2.89	3	1	61.09	Suitable
**ISL-11**	272.2	2.98	3	1	46.53	Suitable
**ISL-12**	285.3	2.95	2	1	40.54	Suitable
**ISL-13**	311.1	4.27	2	1	37.3	Suitable
**ISL-14**	276.2	2.81	3	2	57.53	Suitable
**ISL-15**	326.1	3.59	3	2	63.32	Suitable
**ISL-16**	332.3	2.84	5	1	64.99	Suitable
**ISL-17**	332.3	2.84	5	1	64.99	Suitable
**ISL-18**	292.3	4.25	2	1	37.3	Suitable

*Results were predicted using the Osiris property explorer software available at http://www.organic-chemistry.org/prog/peo/. log *P*: partition coefficient.

Pharmacokinetics, including the absorption, distribution, metabolism and excretion, are essential to determining the therapeutic promise of a lead compound. All the ISL analogues were evaluated by PreADMET server. As shown in Table 3, all the ISL analogues have a plasma protein binding rate of more than 90% with lower blood–brain barrier penetration. The results of human intestinal absorption (HIA), CaCo-2 cell permeability, MDCK cell permeability and skin permeability indicated that ISL analogues have moderate absorption properties through the small intestine. We also predicted the metabolism of ISL analogues through the PreADMET server. As illustrated in Table 4, all the ISL analogues exhibited no inhibitory effect on CYP_2D6 and CYP_3A4 substrate, while CYP_2C19 and CYP_2C9 showed a certain inhibitory effect. For P-glycoprotein (Pgp), most compounds showed promise as an inhibitor except ISL, **ISL-5** and **ISL-7**.

**Table 3:** Absorption and distribution properties of **ISL-1** to **ISL-18** and **ISL**

Comp.	Absorption				Distribution	
	Human intestinal absorption (HIA %)	CaCo-2 cell permeability (nm/s)	MDCK cell permeability (nm/s)	Skin permeability (log kp, cm/h)	Plasma protein binding (%)	Blood–brain barrier penetration (c.brain/c.blood)
**ISL**	88.30	20.15	21.54	–3.28	98.25	1.13
**ISL-1**	95.82	29.44	163.15	−2.37	97.32	1.52
**ISL-2**	95.92	31.59	135.64	−2.29	97.84	3.39
**ISL-3**	95.82	28.15	56.86	−2.60	98.29	1.94
**ISL-4**	96.56	28.42	0.78	−2.34	100.00	3.54
**ISL-5**	93.43	19.65	16.63	−2.97	96.44	0.41
**ISL-6**	95.82	30.28	13.62	−2.60	98.08	2.63
**ISL-7**	93.43	19.84	27.84	−2.98	97.28	0.32
**ISL-8**	95.92	31.55	72.27	−2.27	98.20	2.80
**ISL-9**	95.93	24.94	0.10	−1.51	93.50	5.13
**ISL-10**	95.79	20.66	51.70	−2.64	92.61	0.09
**ISL-11**	95.42	33.76	32.53	−2.60	93.71	0.74
**ISL-12**	95.83	42.02	22.39	−2.64	91.98	1.92
**ISL-13**	96.65	30.05	60.54	−2.43	100.00	6.39
**ISL-14**	92.56	16.28	3.22	−2.90	98.80	1.77
**ISL-15**	94.83	16.99	0.09	−2.90	91.68	1.62
**ISL-16**	95.98	39.81	0.10	−2.89	90.21	0.06
**ISL-17**	95.98	39.03	0.05	−2.92	90.88	0.07
**ISL-18**	96.66	31.47	47.11	−2.31	99.41	3.21

Results were predicted using the server https://preadmet.bmdrc.kr/adme.

**Table 4:** Metabolism of **ISL-1** to **ISL-18** and **ISL** via hepatic microsomal isoforms

Comp.	CYP_2C19 Inhibition	CYP_2C9 inhibition	CYP_2D6 inhibition	CYP_2D6 substrate	CYP_3A4 inhibition	CYP_3A4 substrate	Pgp_inhibition
**ISL**	Inhibitor	Inhibitor	Non-inhibitor	Non-inhibitor	Inhibitor	Non-inhibitor	Non-inhibitor
**ISL-1**	Inhibitor	Inhibitor	Non-inhibitor	Non-inhibitor	Non-inhibitor	Non-inhibitor	Inhibitor
**ISL-2**	Inhibitor	Inhibitor	Non-inhibitor	Non-inhibitor	Non-inhibitor	Non-inhibitor	Inhibitor
**ISL-3**	Inhibitor	Inhibitor	Non-inhibitor	Non-inhibitor	Non-inhibitor	Non-inhibitor	Inhibitor
**ISL-4**	Inhibitor	Inhibitor	Non-inhibitor	Non-inhibitor	Non-inhibitor	Non-inhibitor	Inhibitor
**ISL-5**	Inhibitor	Inhibitor	Non-inhibitor	Non-inhibitor	Non-inhibitor	Non-inhibitor	Non-inhibitor
**ISL-6**	Inhibitor	Inhibitor	Non-inhibitor	Non-inhibitor	Non-inhibitor	Non-inhibitor	Inhibitor
**ISL-7**	Inhibitor	Inhibitor	Non-inhibitor	Non-inhibitor	Non-inhibitor	Non-inhibitor	Non-inhibitor
**ISL-8**	Inhibitor	Inhibitor	Non-inhibitor	Non-inhibitor	Non-inhibitor	Non-inhibitor	Inhibitor
**ISL-9**	Inhibitor	Inhibitor	Non-inhibitor	Non-inhibitor	Non-inhibitor	Non-inhibitor	Inhibitor
**ISL-10**	Inhibitor	Inhibitor	Non-inhibitor	Non-inhibitor	Non-inhibitor	Non-inhibitor	Inhibitor
**ISL-11**	Inhibitor	Inhibitor	Non-inhibitor	Non-inhibitor	Non-inhibitor	Non-inhibitor	Inhibitor
**ISL-12**	Non-inhibitor	Inhibitor	Non-inhibitor	Non-inhibitor	Non-inhibitor	Non-inhibitor	Inhibitor
**ISL-13**	Inhibitor	Inhibitor	Non-inhibitor	Non-inhibitor	Non-inhibitor	Non-inhibitor	Inhibitor
**ISL-14**	Inhibitor	Inhibitor	Non-inhibitor	Non-inhibitor	Inhibitor	Non-inhibitor	Inhibitor
**ISL-15**	Inhibitor	Inhibitor	Non-inhibitor	Non-inhibitor	Non-inhibitor	Non-inhibitor	Inhibitor
**ISL-16**	Inhibitor	Inhibitor	Non-inhibitor	Non-inhibitor	Inhibitor	Weakly	Inhibitor
**ISL-17**	Inhibitor	Inhibitor	Non-inhibitor	Non-inhibitor	Inhibitor	Weakly	Inhibitor
**ISL-18**	Inhibitor	Inhibitor	Non-inhibitor	Non-inhibitor	Non-inhibitor	Non-inhibitor	Inhibitor

Results were predicted using the server https://preadmet.bmdrc.kr/adme.

## Discussion and conclusion

Naturally derived compounds are of great significance in drug discovery [32]. In the present study, a series of ISL analogues were designed and synthesized. Among all 18 ISL analogues, **ISL-17** showed the best inhibitory activity against two different human gastric cancer cells *in vitro*, including SGC-7901 and BGC-823 cells. Detailed antitumor mechanism studies show that **ISL-17** could induce G2/M cell cycle arrest and apoptosis in SGC-7901 and BGC-823 cells. **ISL-17** could also induce autophagy and influence the generation of ROS in SGC-7901 cells. Furthermore, we found that **ISL-17** may exert its antitumor effect through PI3K/AKT/mTOR pathway in SGC-7901 cells. Drug-likeness prediction indicated that most ISL analogues obey *Lipinski's rule of five* and *Veber rules* and have suitable ADME properties, especially for **ISL-17**.

Actually, previous studies have shown that ISL has therapeutic potential in the treatment of human gastric cancer. For example, Zhang et al. [11] found that ISL could inhibit proliferation and metastasis in MKN28 gastric cancer cells. Ma et al. [12] revealed that ISL could induce apoptosis in MGC-803 gastric cancer cells. However, in spite of its broad antitumor activities, low bioavailability of ISL [17] was a problem that limits its wider application. To our knowledge, little research has focused on structural modification based on ISL for the treatment of gastric cancer. Therefore, the present study presented and tested **ISL-17**, a new compound with potential anti-gastric cancer activity, which showed better properties than ISL in some aspects. Admittedly, some limitations of the present study must also be acknowledged. The results of the present study come from *in vitro* experiments only, which means that more studies are required in the future to reveal its detailed antitumor mechanisms *in vivo*. In conclusion, the discovery of a novel ISL analog (**ISL-17**) has enriched the anti-gastric cancer molecular library and is worthy of further development.

## Supplementary Material

Supplementary Information

## Abbreviations

DMSO: dimethyl sulfoxide
ESI: electrospray ion source
HBA: hydrogen bond acceptor
HBD: hydrogen bond donor
HIA: human intestinal absorption
HRMS: high-resolution mass spectra
ISL: isoliquiritigenin
MW: molecular weight
NAC: *N*-acetyl cysteine
NMR: nuclear magnetic resonance
PBS: phosphate buffered saline
PSA: polar surface area
ROS: reactive oxygen species
TLC: thin layer chromatography
