# Supplementary material for: Discovery of novel isoliquiritigenin analogue ISL-17 as a potential anti-gastric cancer agent
Source: Biosci Rep. 2020 Jun 19;40(6):BSR20201199. doi: 10.1042/BSR20201199 (PMC7306486; doi:10.1042/BSR20201199)
Supplement: Supplementary Information [file BSR-2020-1199_supp.pdf]

Supplementary Information

Discovery of novel Isoliquiritigenin analogue ISL-17 as a potential anti-gastric cancer agent

Fengchang Huang[1], Jin Wang[2], Yi Xu[3], Yunfei Zhang[1], Ning Xu[1], Liang Yin[1,]*

[1] Department of Oncology, the First Affiliated Hospital of Kunming Medical University, Kunming 650032, Yunnan Province, China

[2] Department of Vascular surgery, the First Affiliated Hospital of Kunming Medical University, Kunming 650032, Yunnan Province, China

[3] Department of Pharmacy, the First Affiliated Hospital of Wenzhou Medical University, Wenzhou 325000, Zhejiang, China

*** Correspondence**

Liang Yin

Department of Oncology, the First Affiliated Hospital of Kunming Medical University, No. 295 Xichang Road, Wuhua District, Kunming 650032, Yunnan Province, China

E-mail: kmumdyl@sina.com

Tel: +86-0871-65324888; **Fax:** +86-0871-65324888

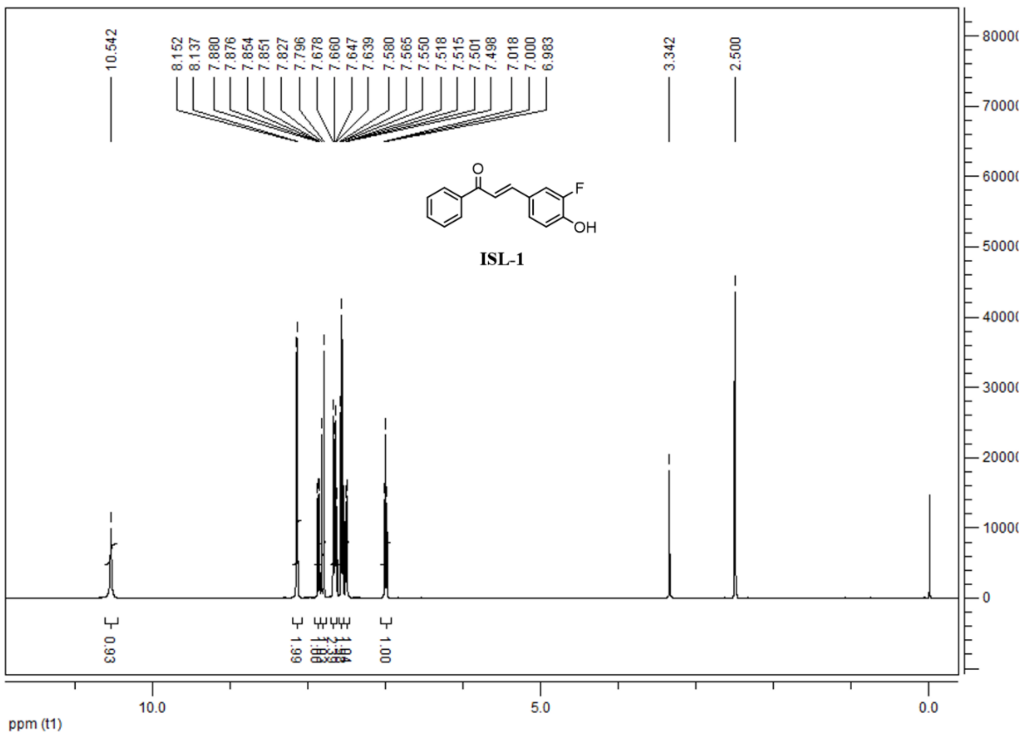

Figure S1. The ^{1}H NMR spectra of compound **ISL-1** (DMSO-d_6, Bruker AVANVEIII 400)

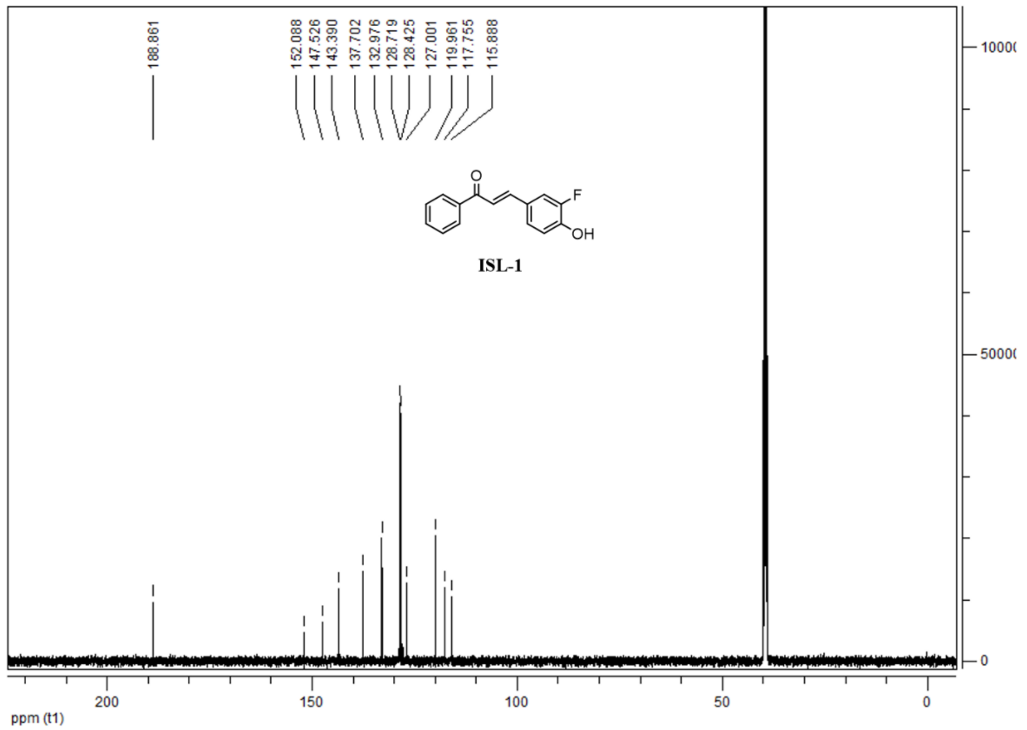

Figure S2. The ^{13}C NMR spectra of compound **ISL-1** (DMSO-d_6, Bruker AVANVEIII 400)

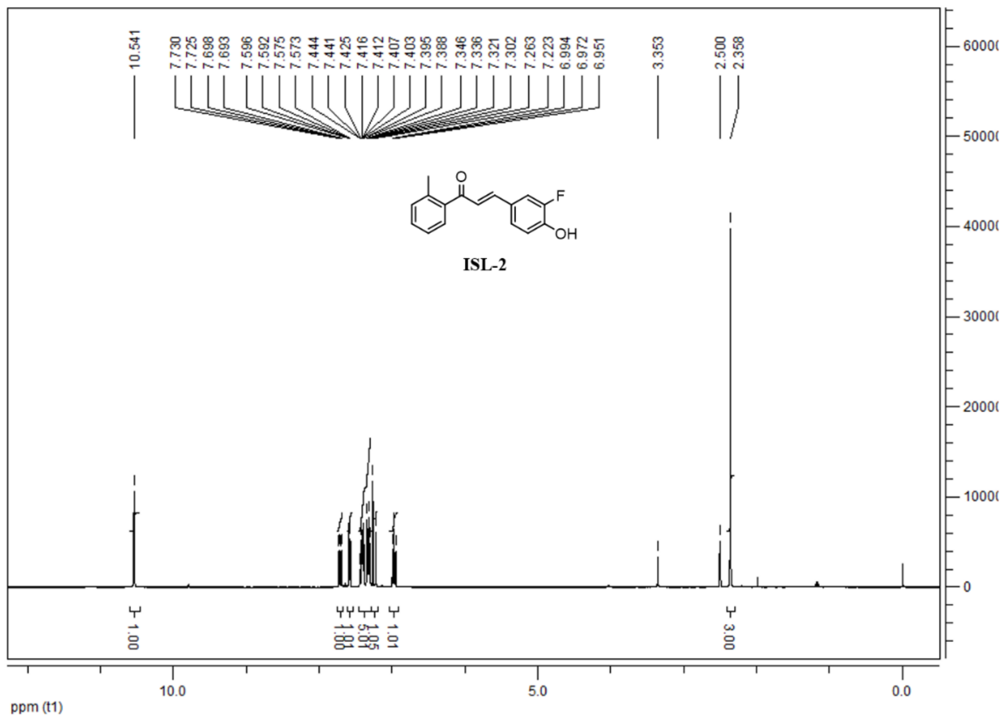

Figure S3. The ^{1}H NMR spectra of compound **ISL-2** (DMSO-d_6, Bruker AVANVEIII 400)

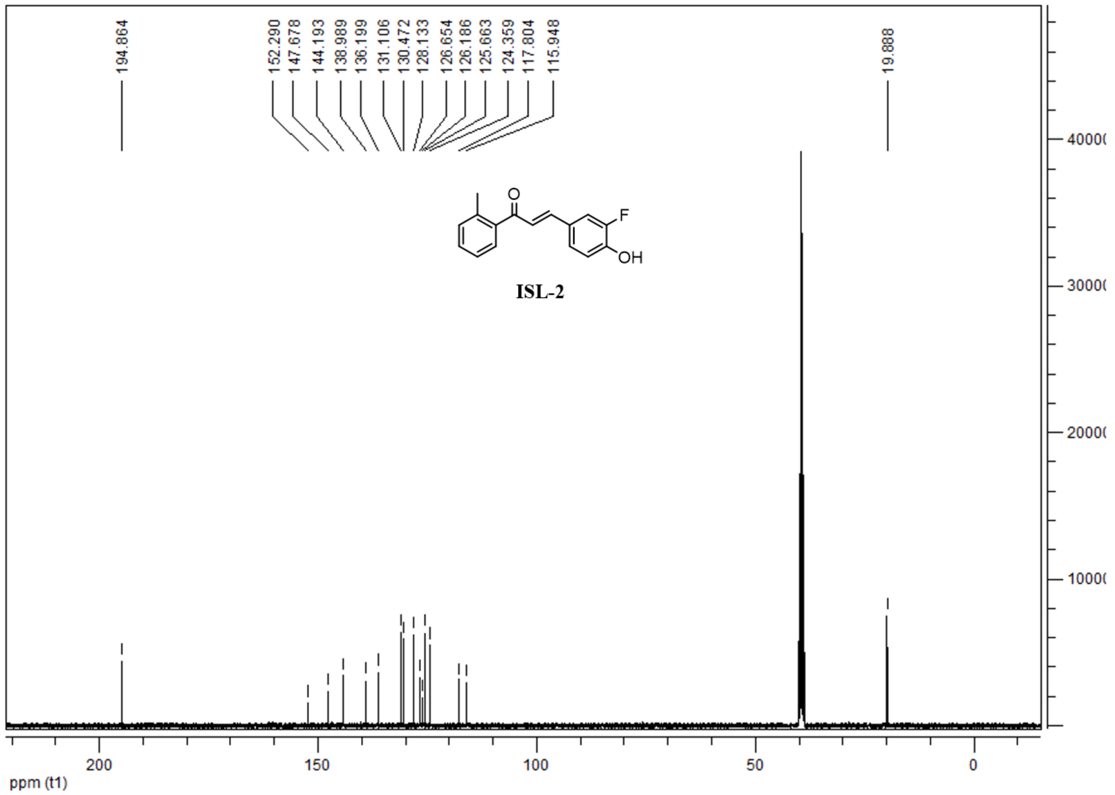

Figure S4. The ^{13}C NMR spectra of compound **ISL-2** (DMSO-d_6, Bruker AVANVEIII 400)

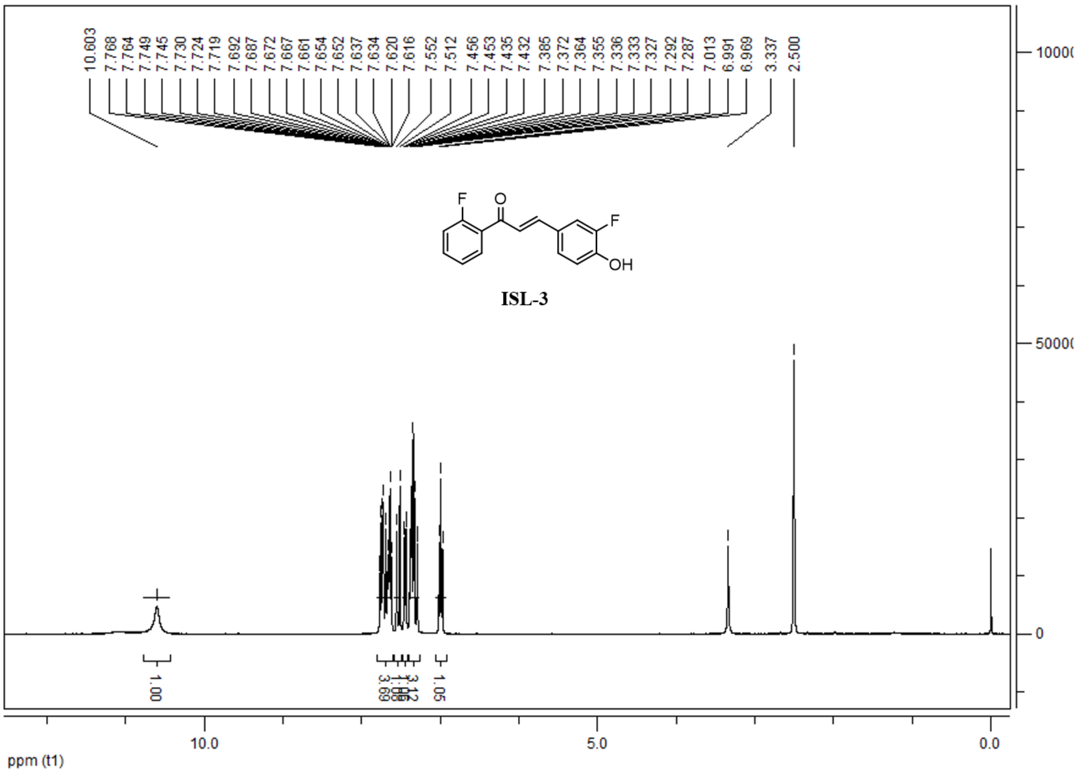

Figure S5. The ^{1}H NMR spectra of compound **ISL-3** (DMSO-d_6, Bruker AVANVEIII 400)

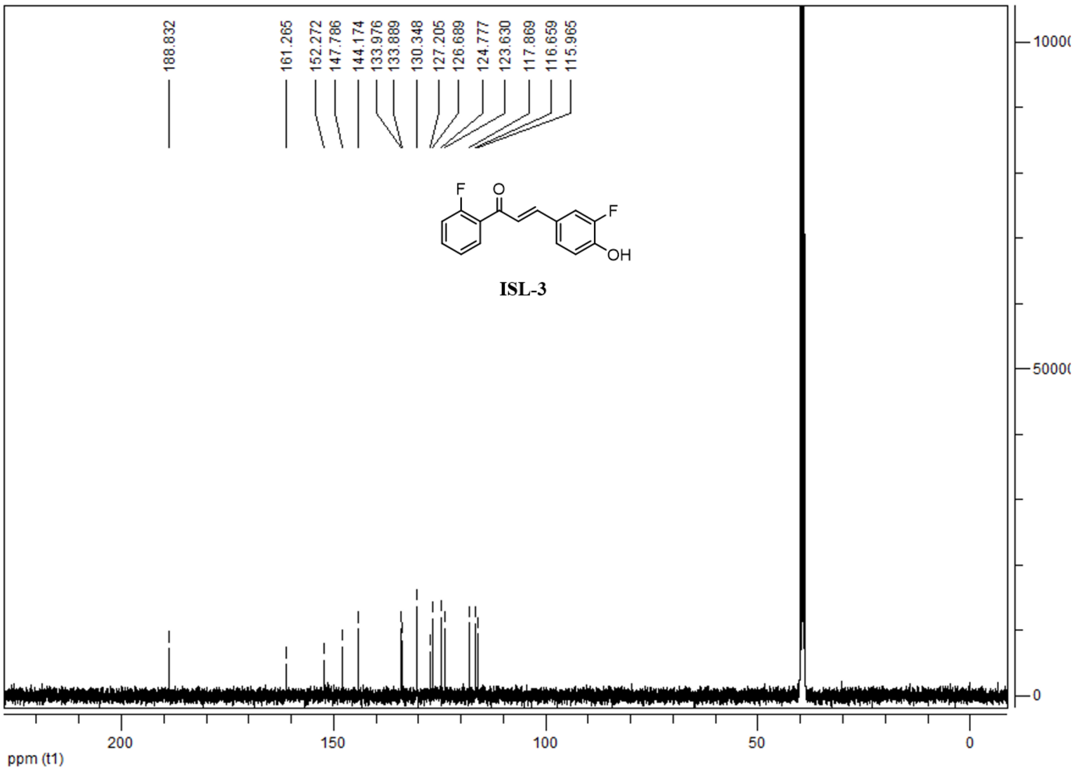

Figure S6. The ^{13}C NMR spectra of compound **ISL-3** (DMSO-d_6, Bruker AVANVEIII 400)

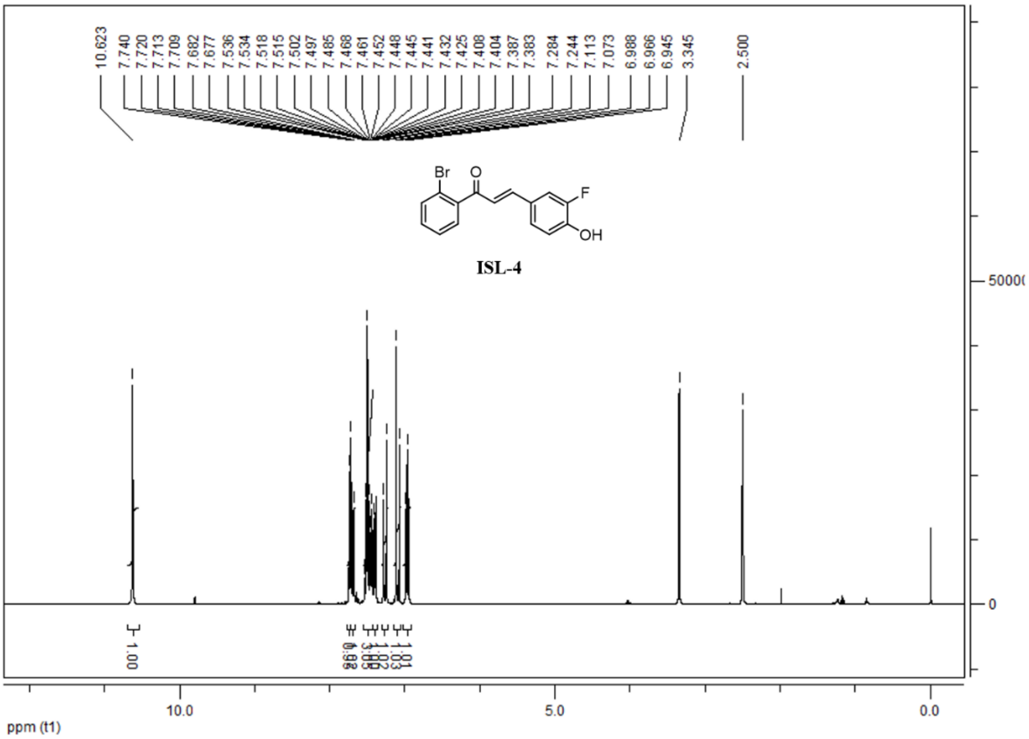

Figure S7. The ^{1}H NMR spectra of compound **ISL-4** (DMSO-d_6, Bruker AVANVEIII 400)

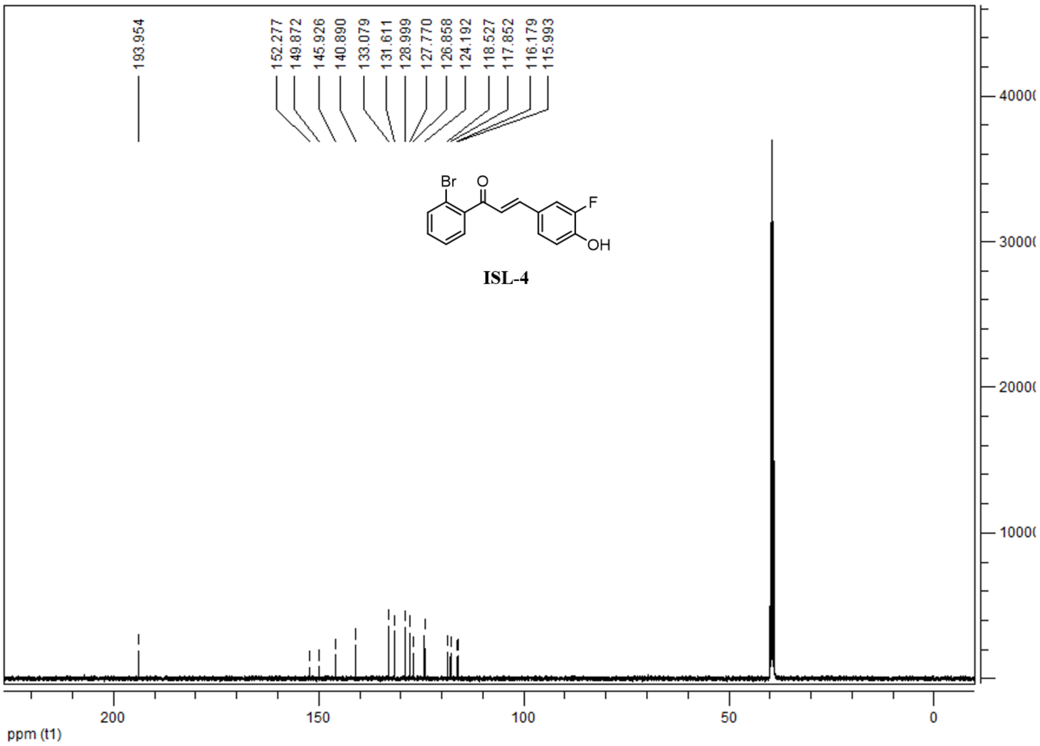

Figure S8. The ^{13}C NMR spectra of compound **ISL-4** (DMSO-d_6, Bruker AVANVEIII 400)

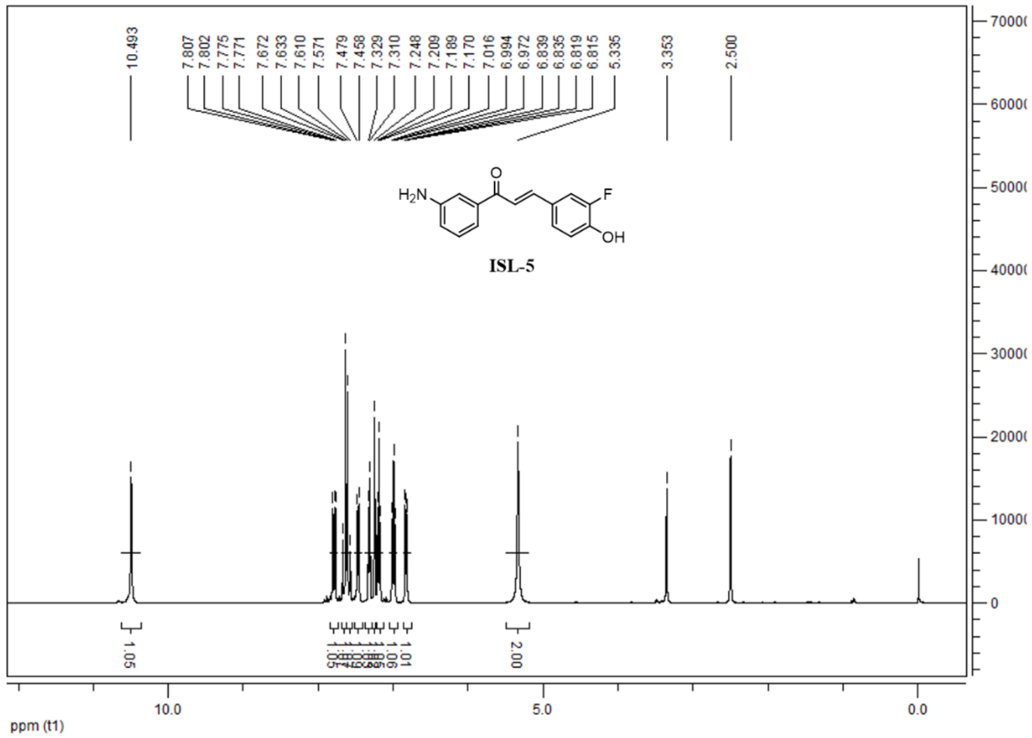

Figure S9. The ^{1}H NMR spectra of compound **ISL-5** (DMSO-d_6, Bruker AVANVEIII 400)

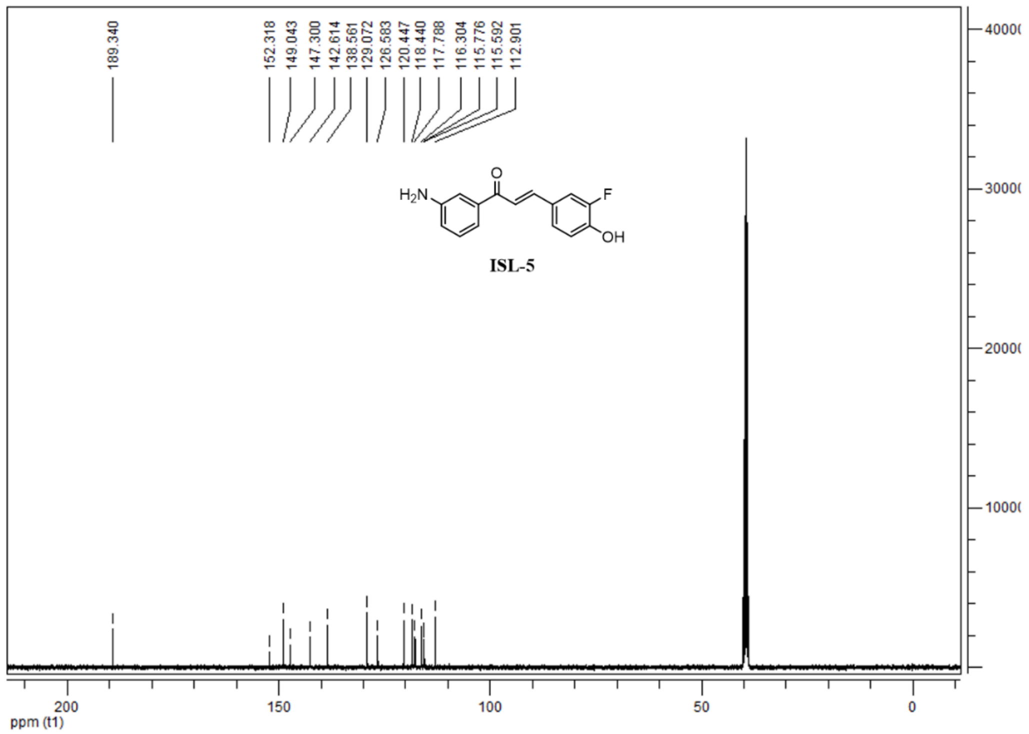

Figure S10. The ^{13}C NMR spectra of compound **ISL-5** (DMSO-d_6, Bruker AVANVEIII 400)

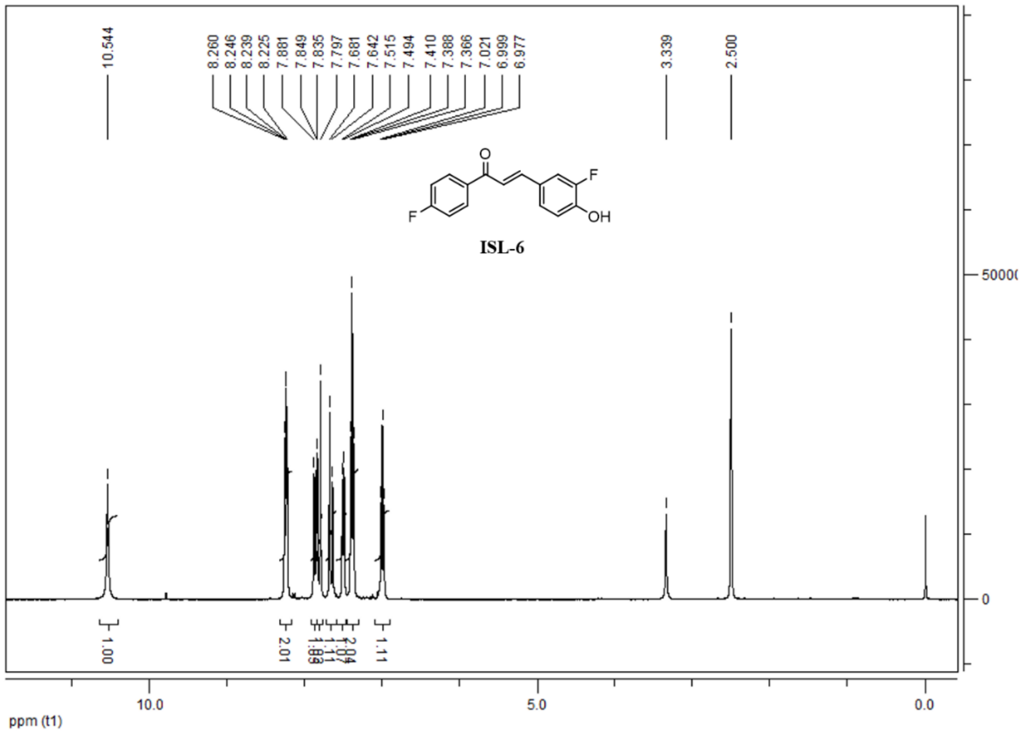

Figure S11. The ^{1}H NMR spectra of compound **ISL-6** (DMSO-d_6, Bruker AVANVEIII 400)

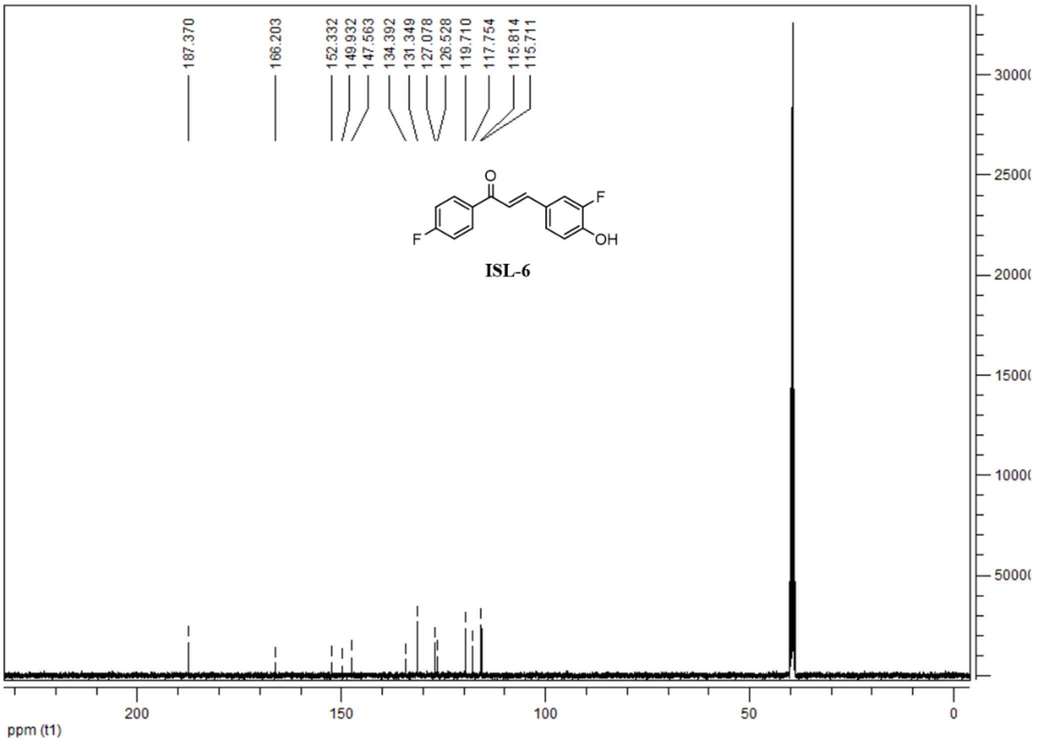

Figure S12. The ^{13}C NMR spectra of compound **ISL-6** (DMSO-d_6, Bruker AVANVEIII 400)

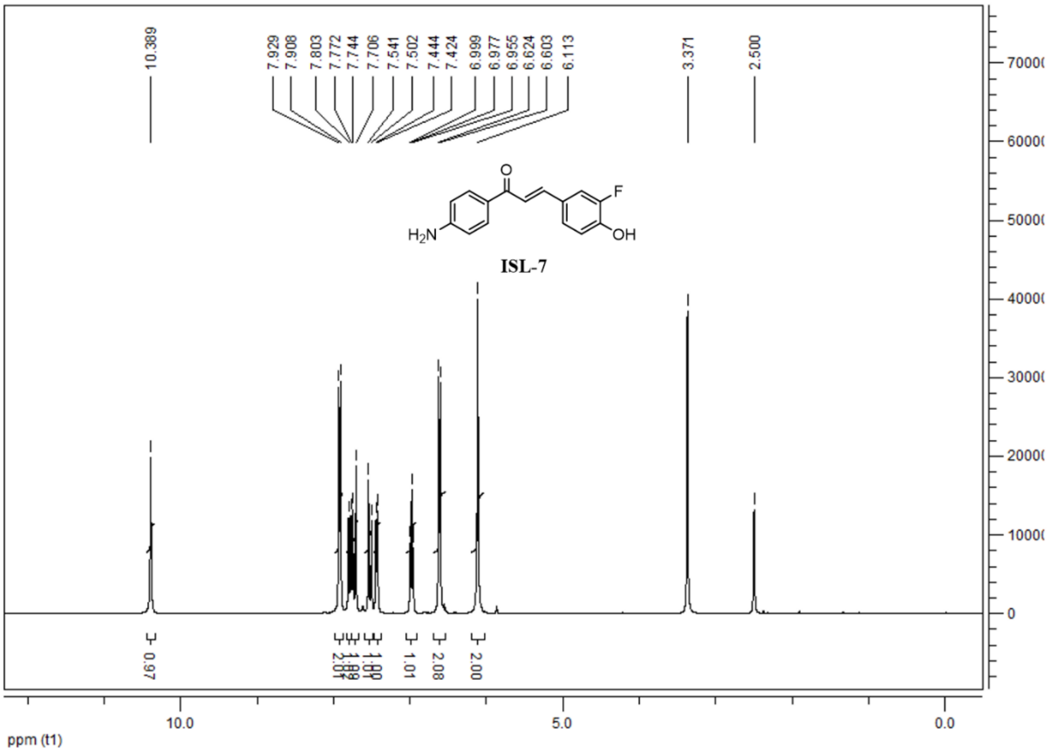

Figure S13. The ^{1}H NMR spectra of compound **ISL-7** (DMSO-d_6, Bruker AVANVEIII 400)

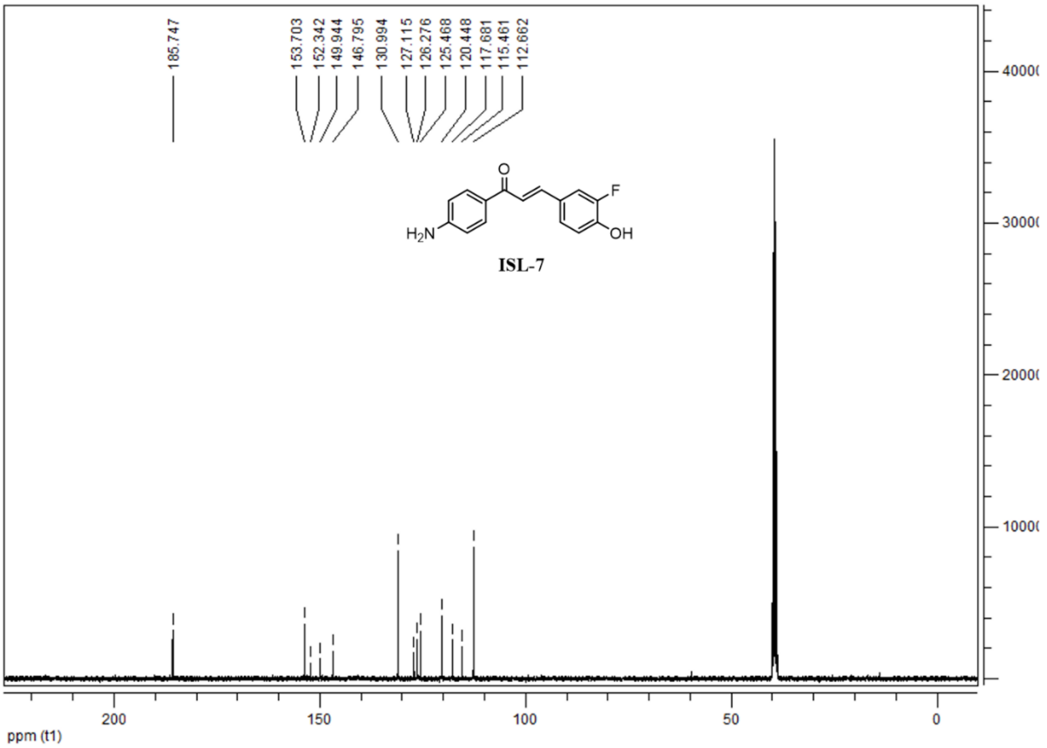

Figure S14. The ^{13}C NMR spectra of compound **ISL-7** (DMSO-d_6, Bruker AVANVEIII 400)

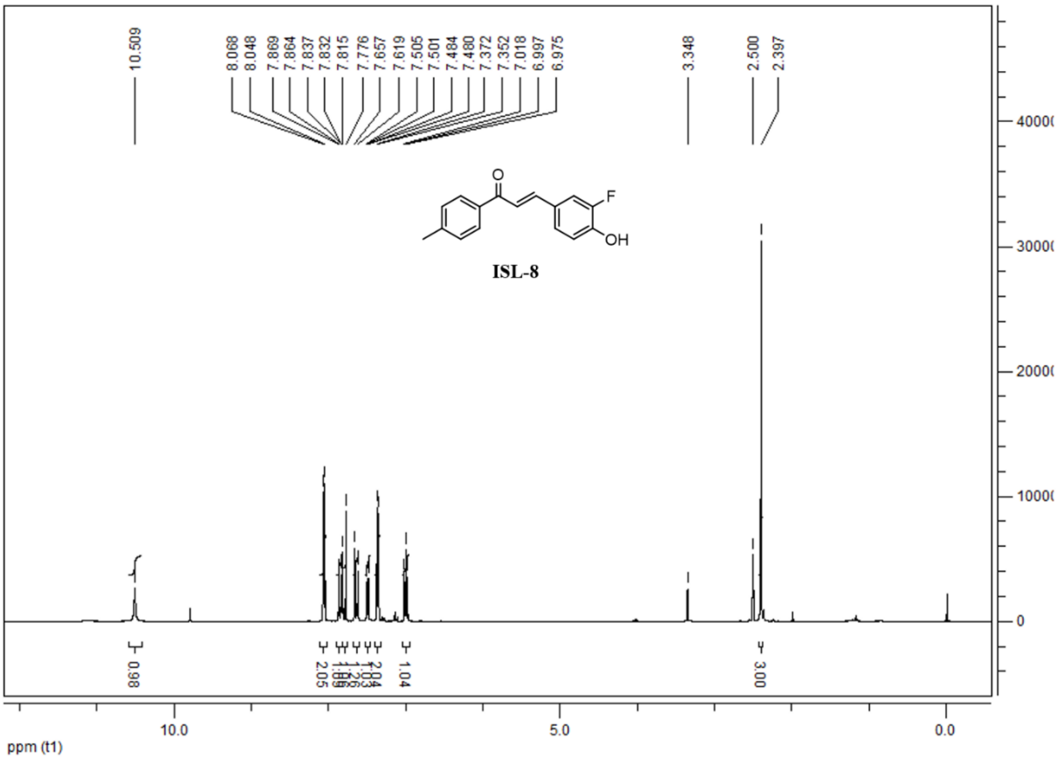

Figure S15. The ^{1}H NMR spectra of compound **ISL-8** (DMSO-d_6, Bruker AVANVEIII 400)

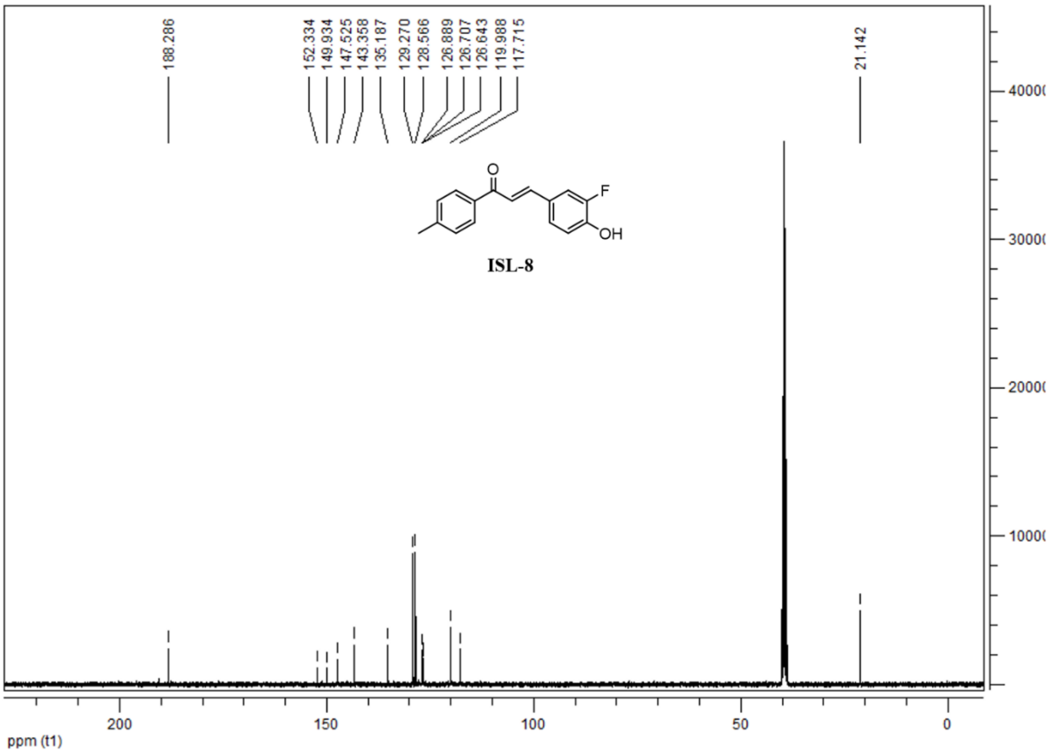

Figure S16. The ^{13}C NMR spectra of compound **ISL-8** (DMSO-d_6, Bruker AVANVEIII 400)

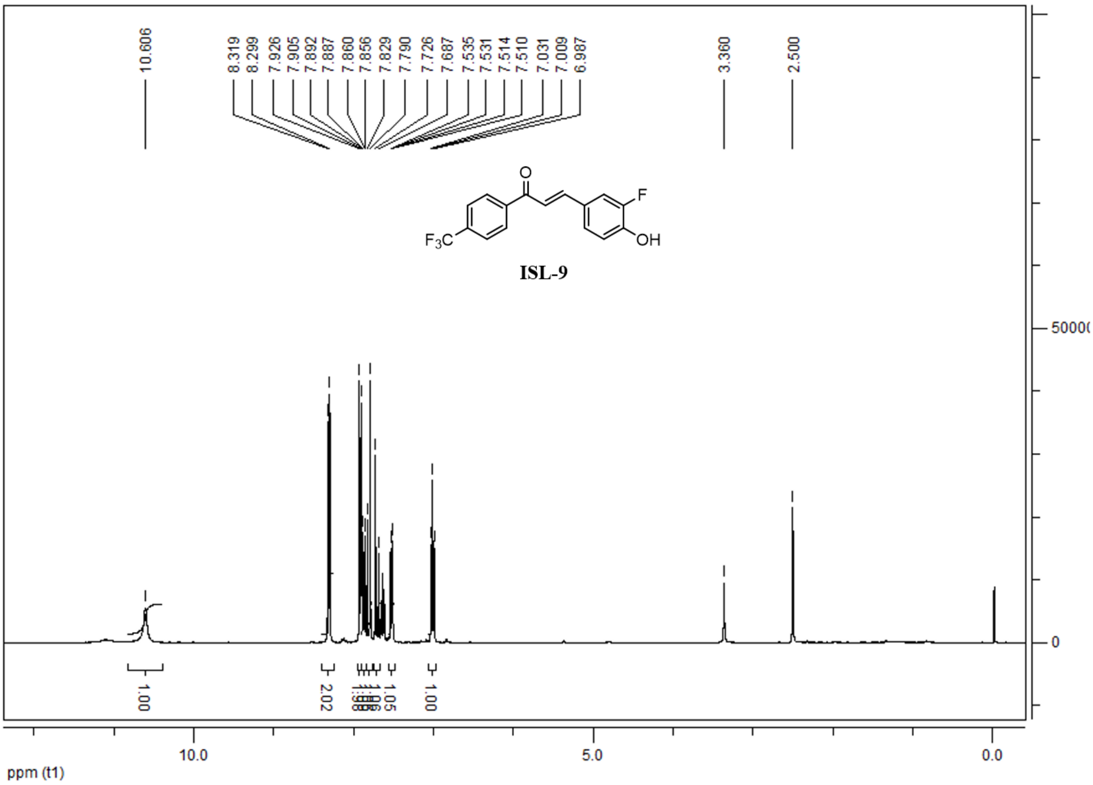

Figure S17. The ^{1}H NMR spectra of compound **ISL-9** (DMSO-d_6, Bruker AVANVEIII 400)

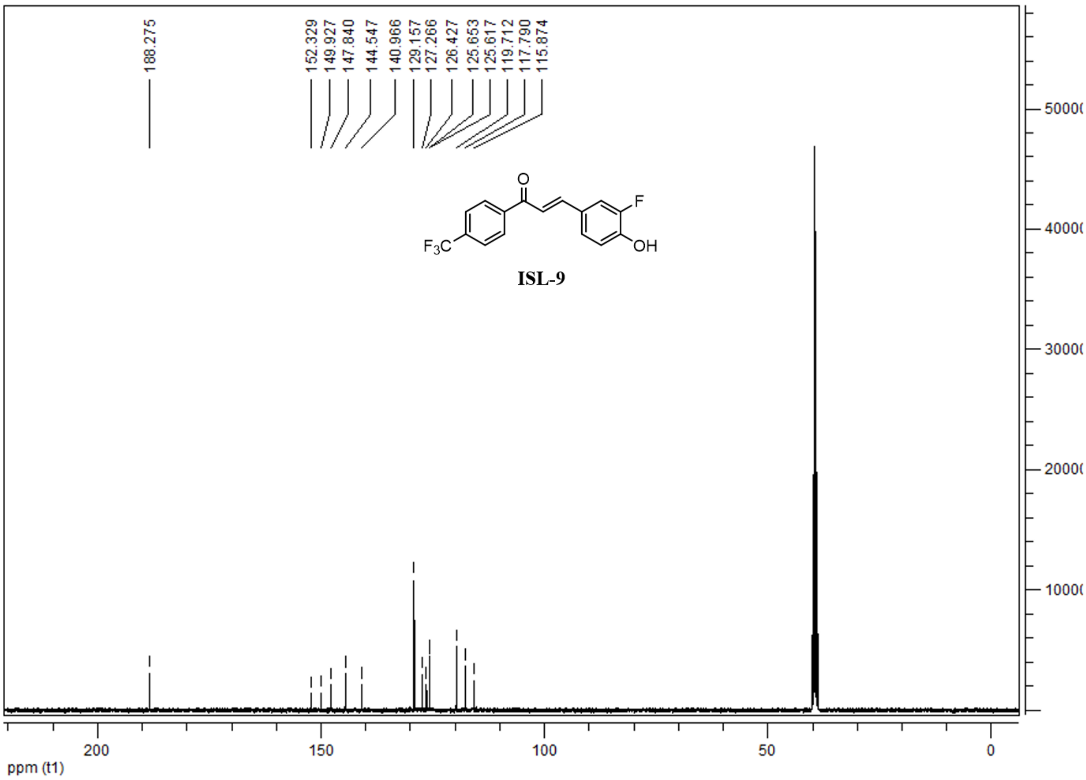

Figure S18. The ^{13}C NMR spectra of compound **ISL-9** (DMSO-d_6, Bruker AVANVEIII 400)

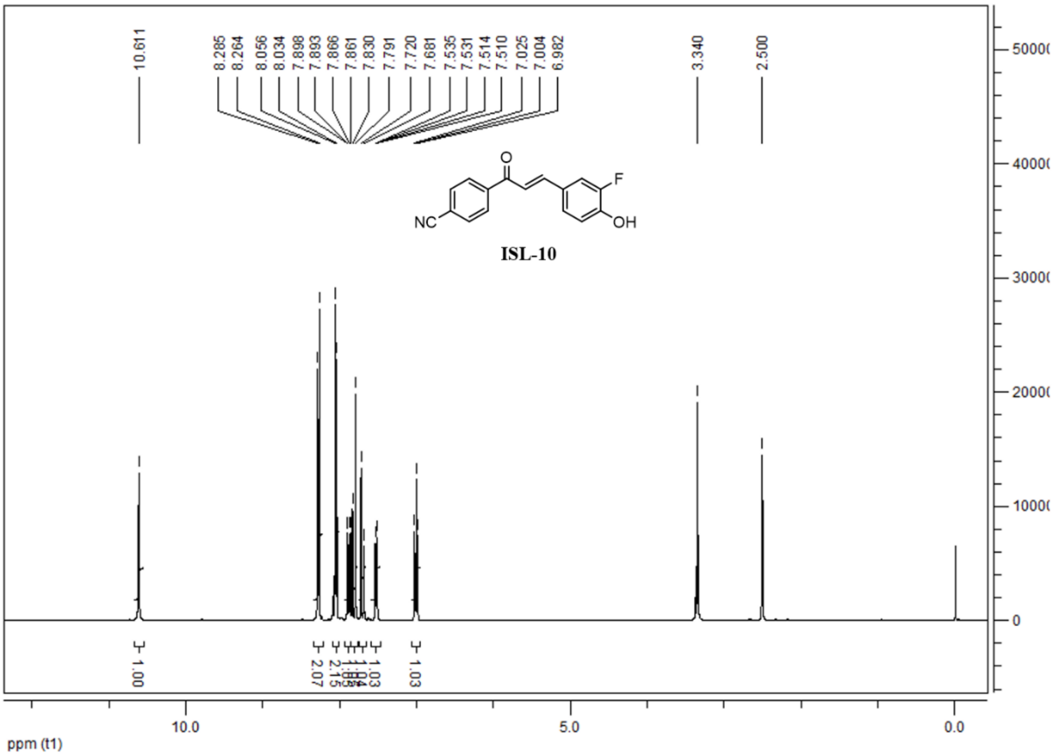

Figure S19. The ^{1}H NMR spectra of compound **ISL-10** (DMSO-d_6, Bruker AVANVEIII 400)

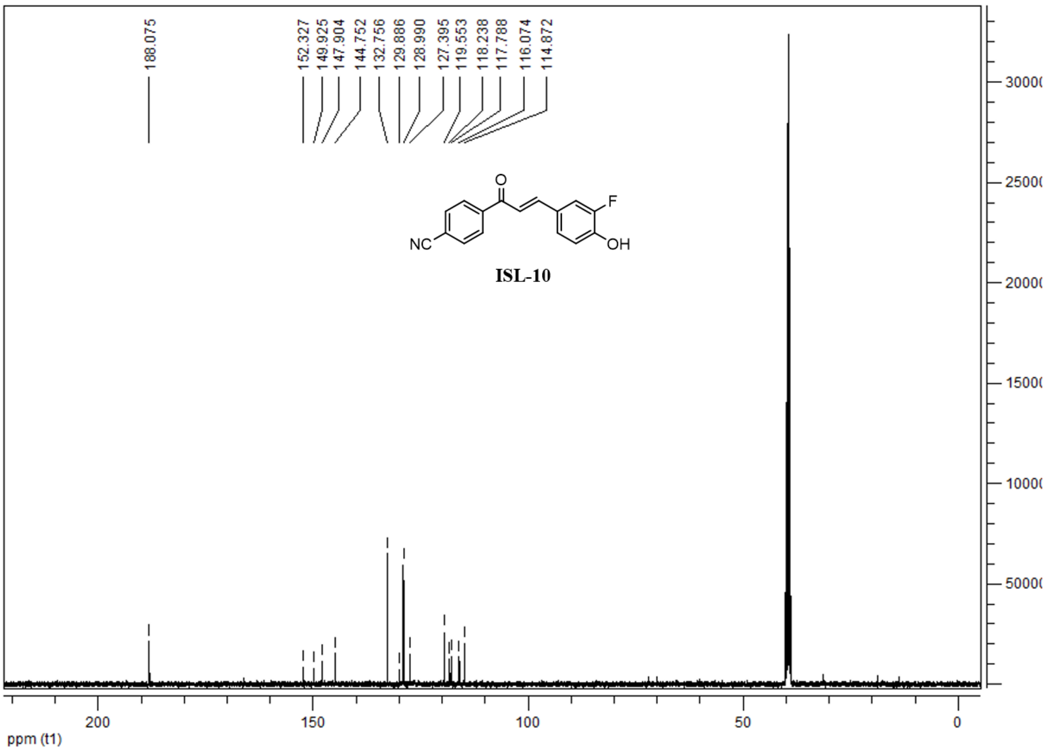

Figure S20. The ^{13}C NMR spectra of compound **ISL-10** (DMSO-d_6, Bruker AVANVEIII 400)

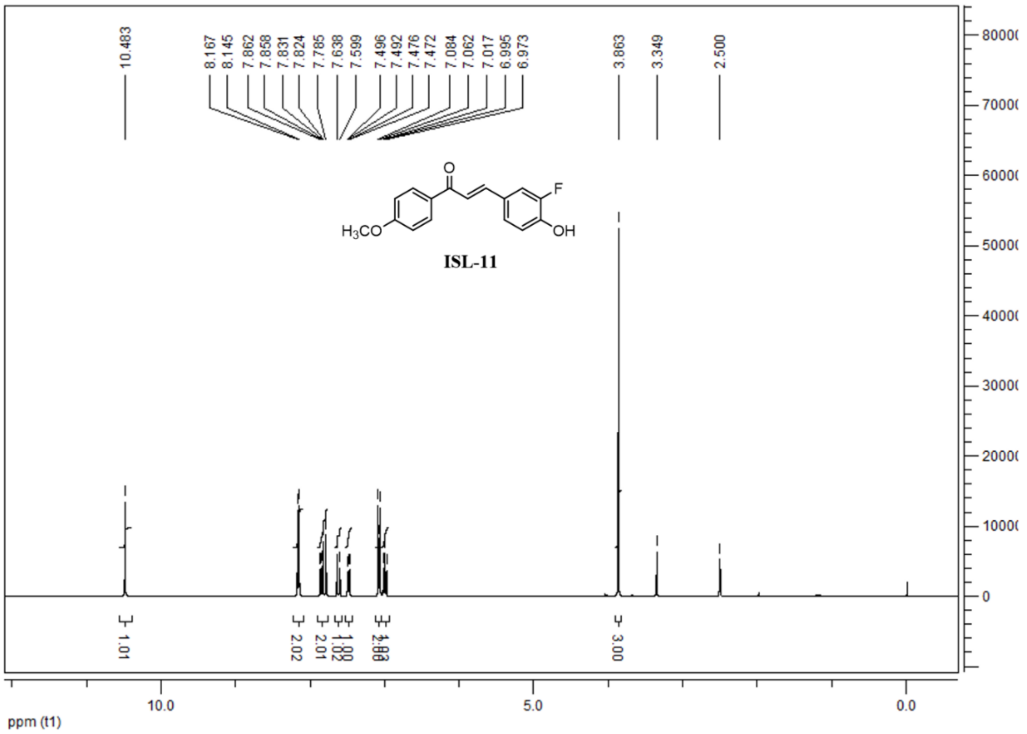

Figure S21. The ^{1}H NMR spectra of compound **ISL-11** (DMSO-d_6, Bruker AVANVEIII 400)

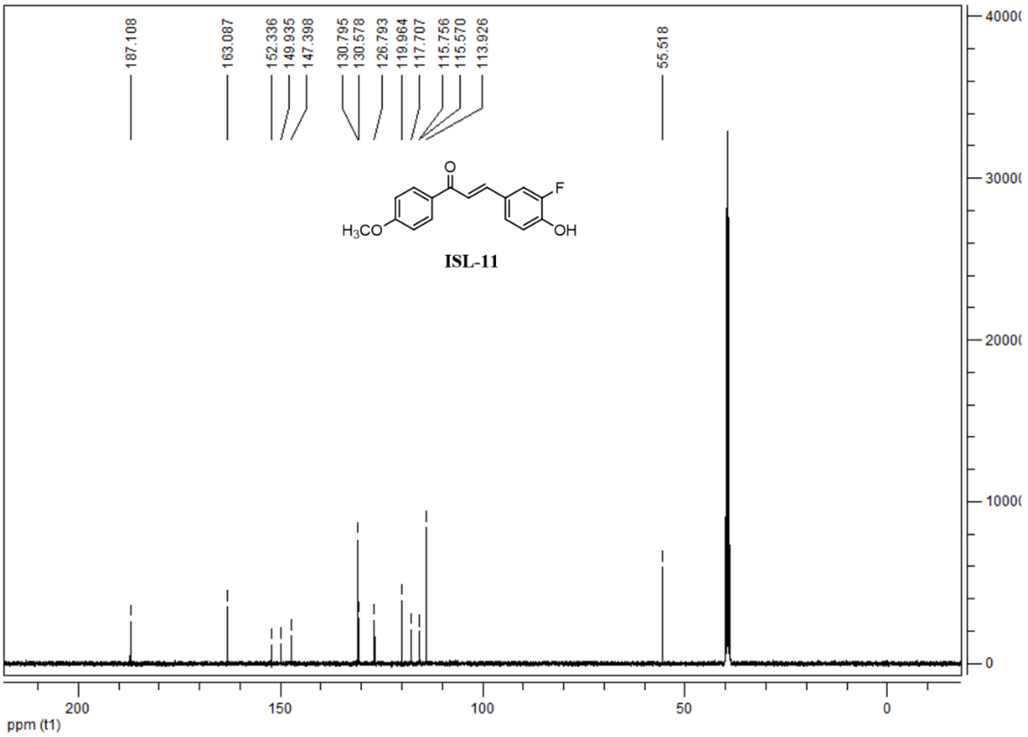

Figure S22. The ^{13}C NMR spectra of compound **ISL-11** (DMSO-d_6, Bruker AVANVEIII 400)

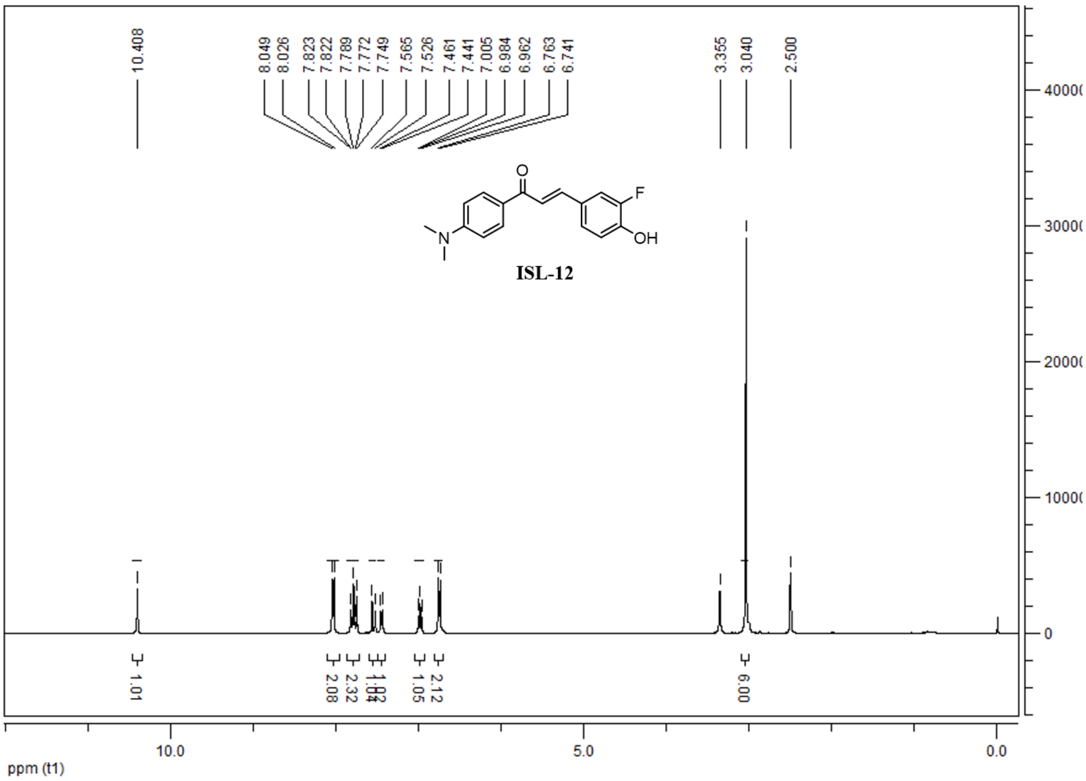

Figure S23. The ^{1}H NMR spectra of compound **ISL-12** (DMSO-d_6, Bruker AVANVEIII 400)

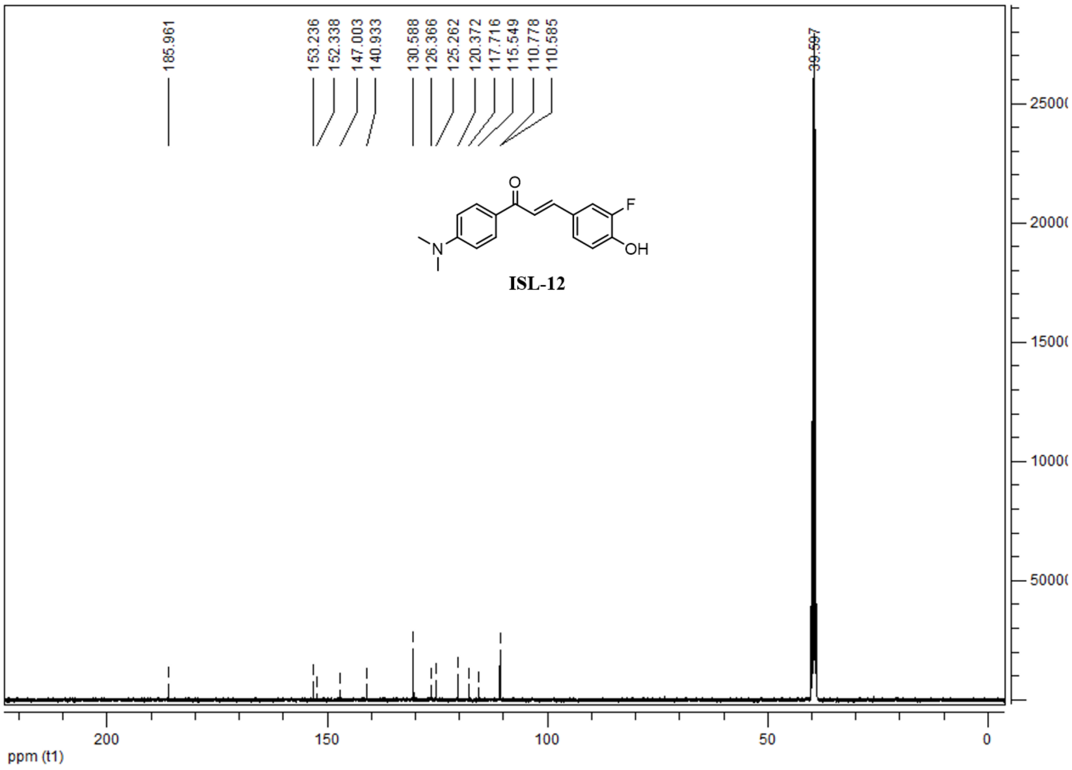

Figure S24. The ^{13}C NMR spectra of compound **ISL-12** (DMSO-d_6, Bruker AVANVEIII 400)

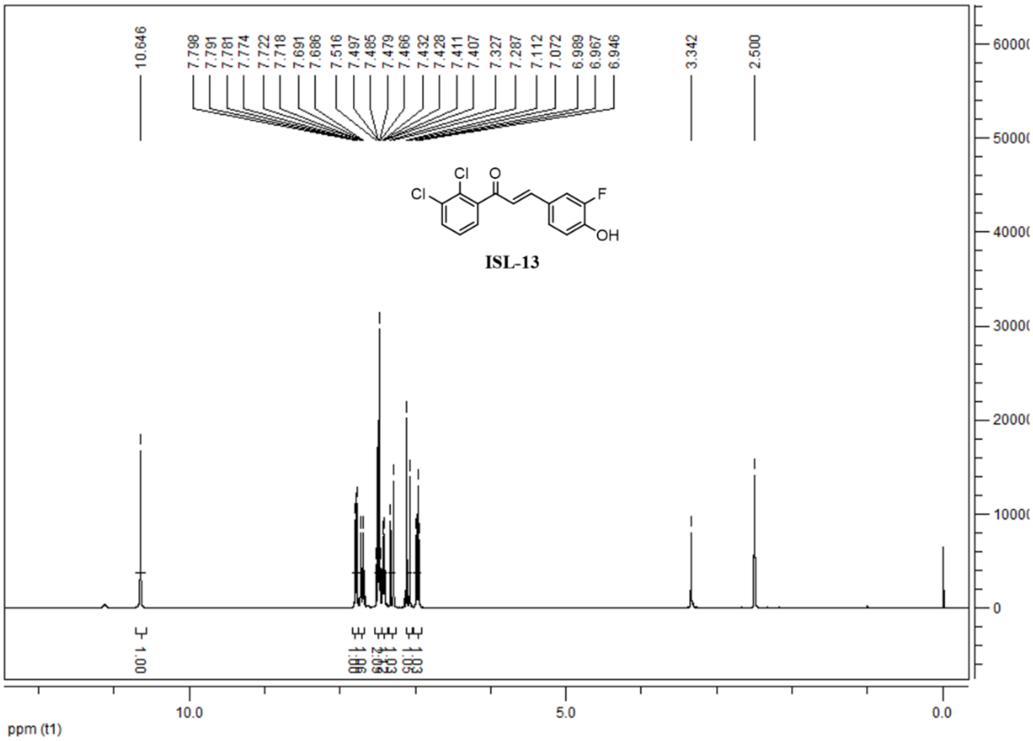

Figure S25. The ^{1}H NMR spectra of compound **ISL-13** (DMSO-d_6, Bruker AVANVEIII 400)

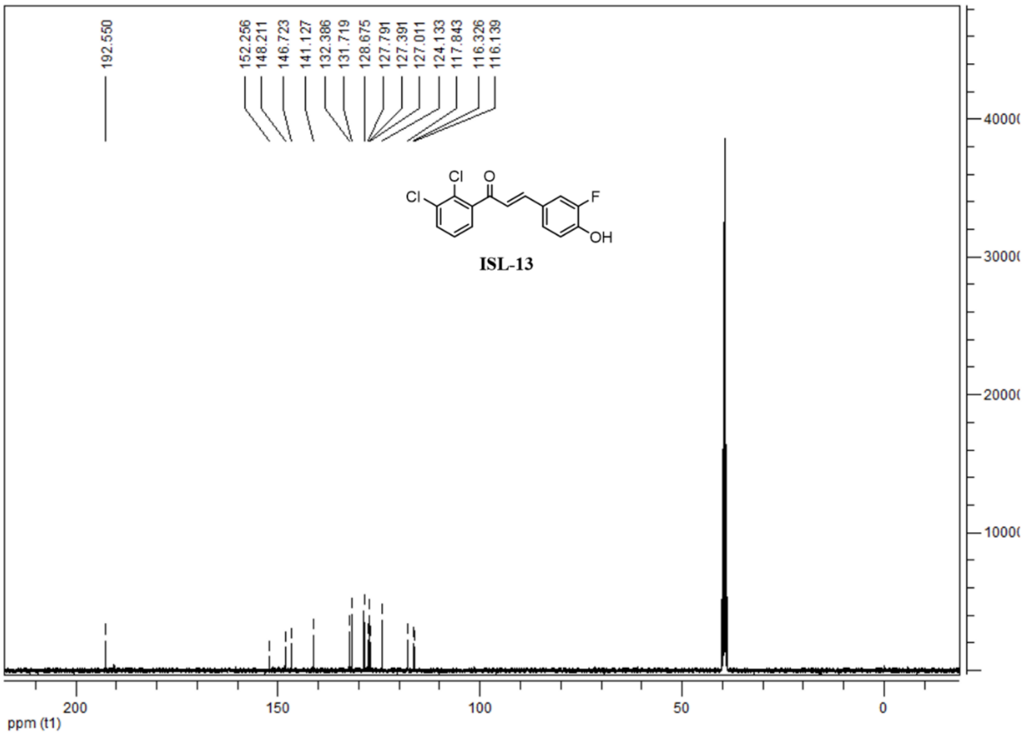

Figure S26. The ^{13}C NMR spectra of compound **ISL-13** (DMSO-d_6, Bruker AVANVEIII 400)

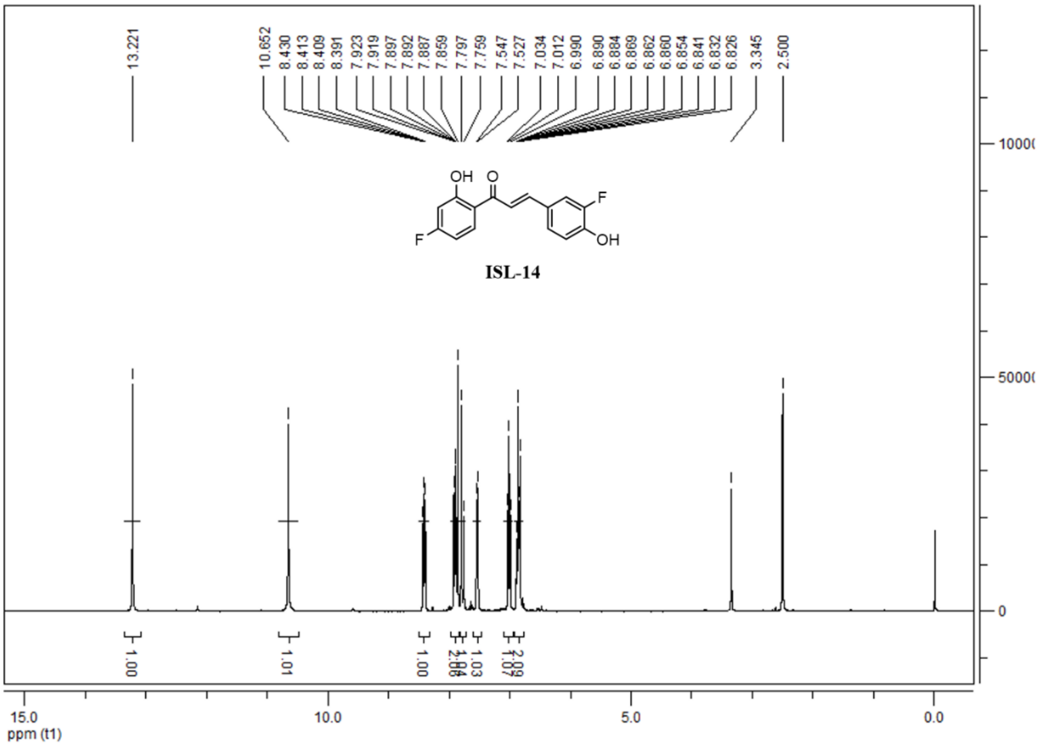

Figure S27. The ^{1}H NMR spectra of compound **ISL-14** (DMSO-d_6, Bruker AVANVEIII 400)

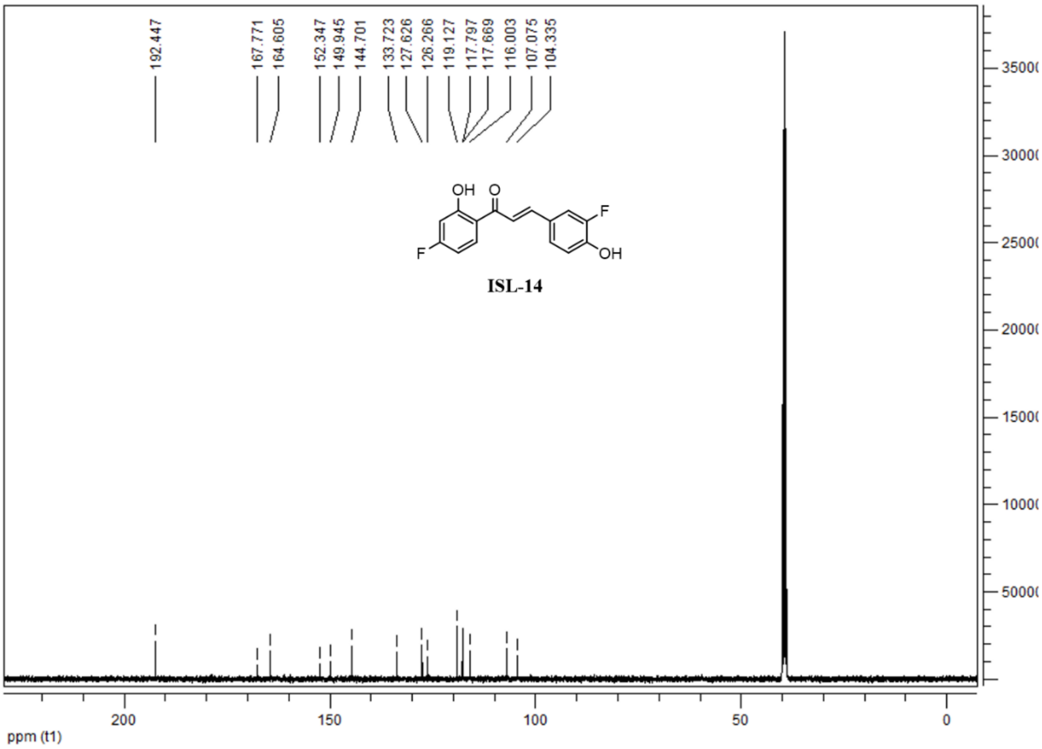

Figure S28. The ^{13}C NMR spectra of compound **ISL-14** (DMSO-d_6, Bruker AVANVEIII 400)

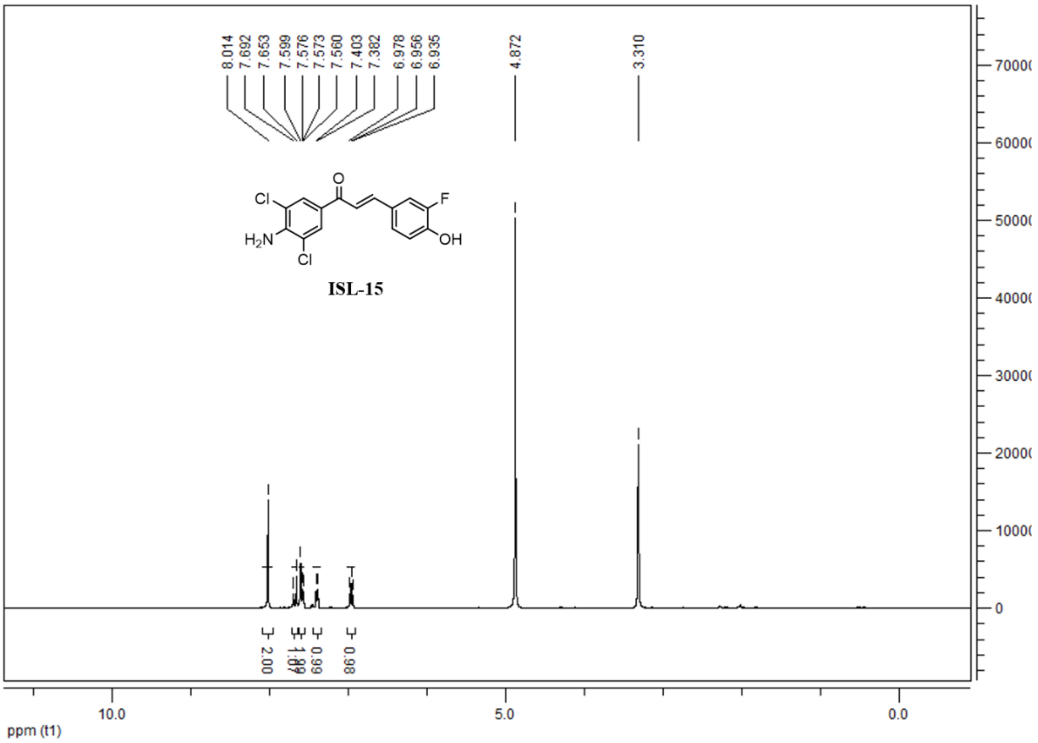

Figure S29. The ^{1}H NMR spectra of compound **ISL-15** (DMSO-d_6, Bruker AVANVEIII 400)

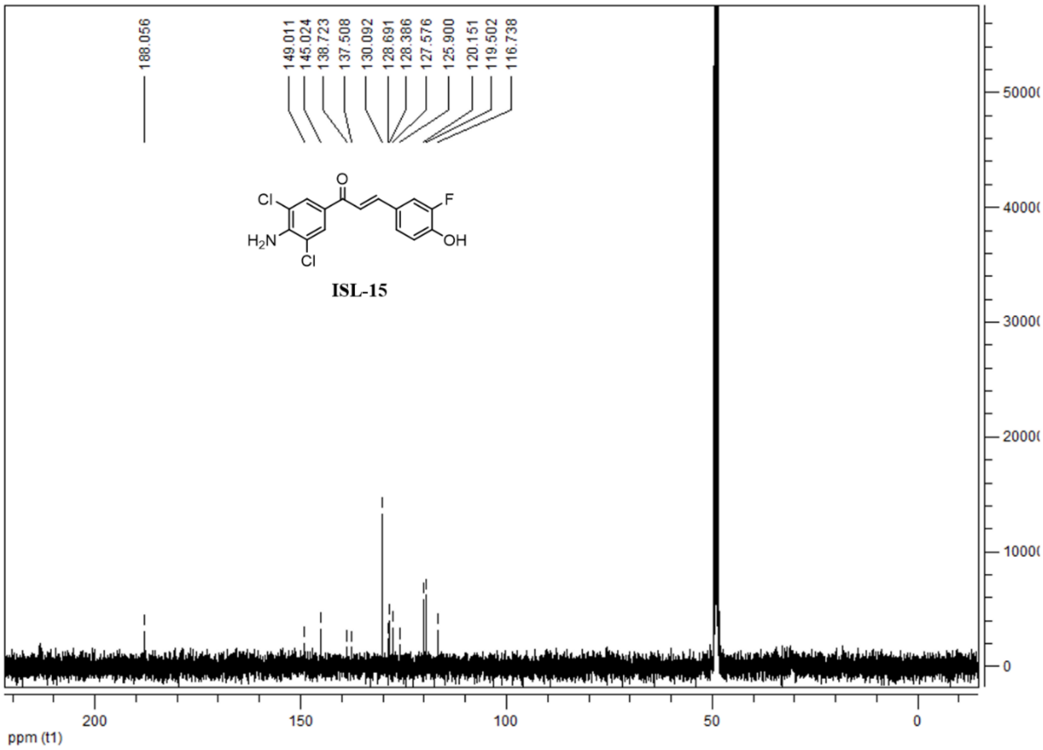

Figure S30. The ^{13}C NMR spectra of compound **ISL-15** (DMSO-d_6, Bruker AVANVEIII 400)

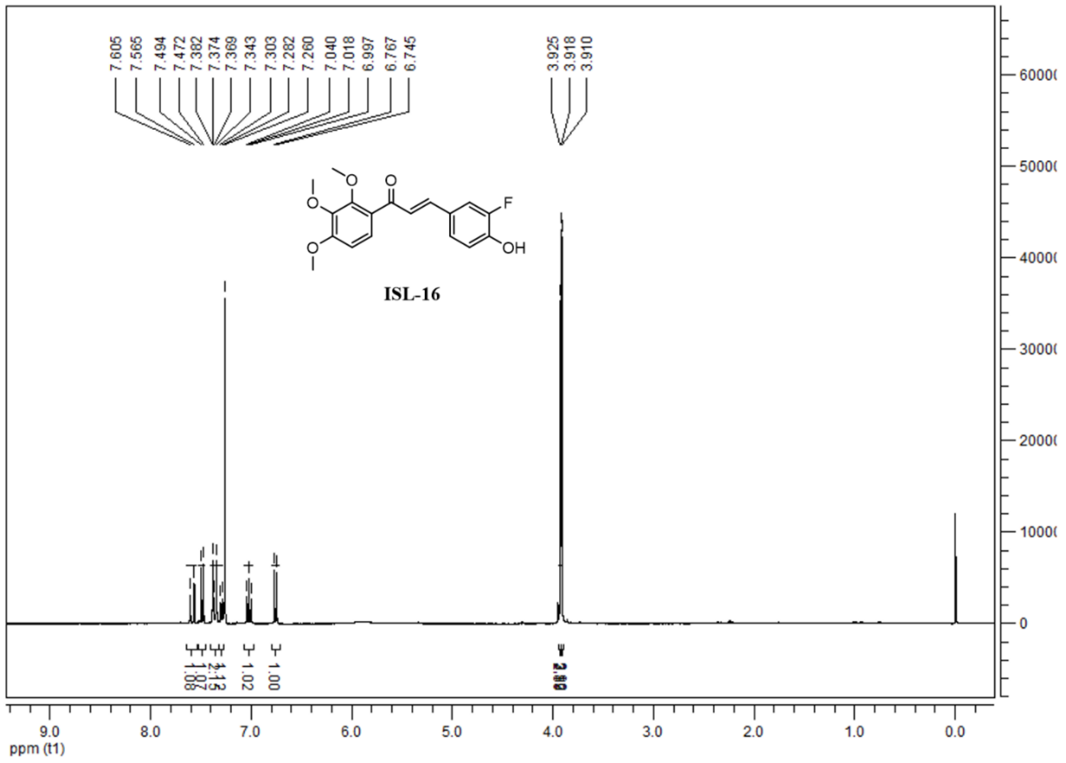

Figure S31. The ^{1}H NMR spectra of compound **ISL-16** (DMSO-d_6, Bruker AVANVEIII 400)

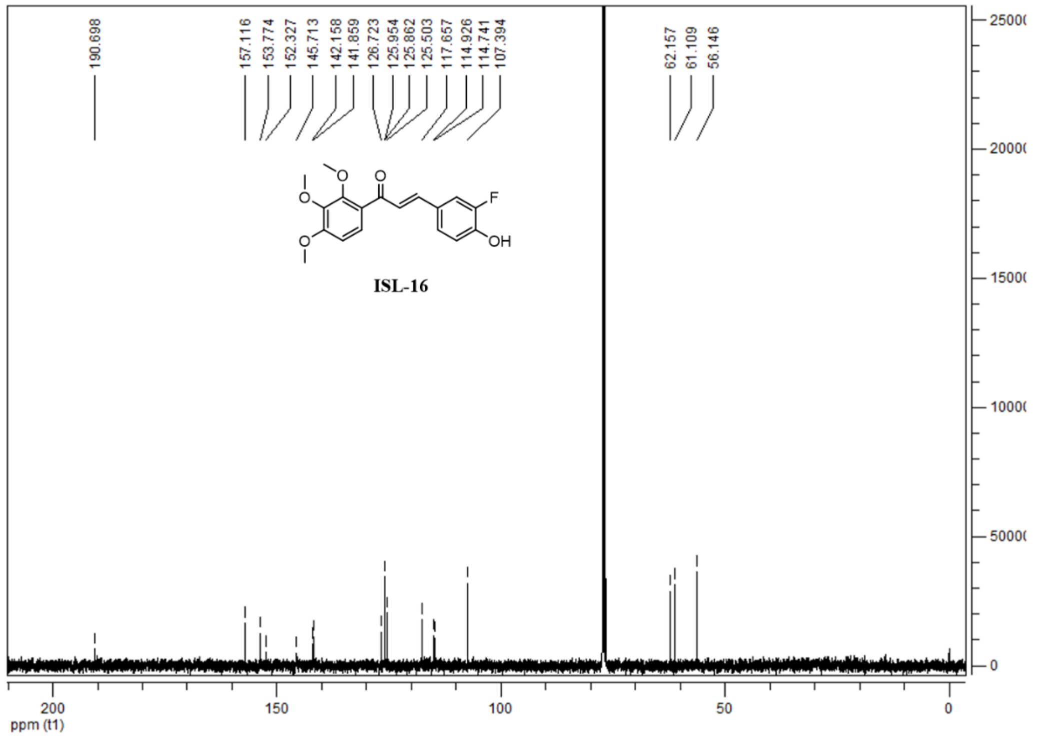

Figure S32. The ^{13}C NMR spectra of compound **ISL-16** (DMSO-d_6, Bruker AVANVEIII 400)

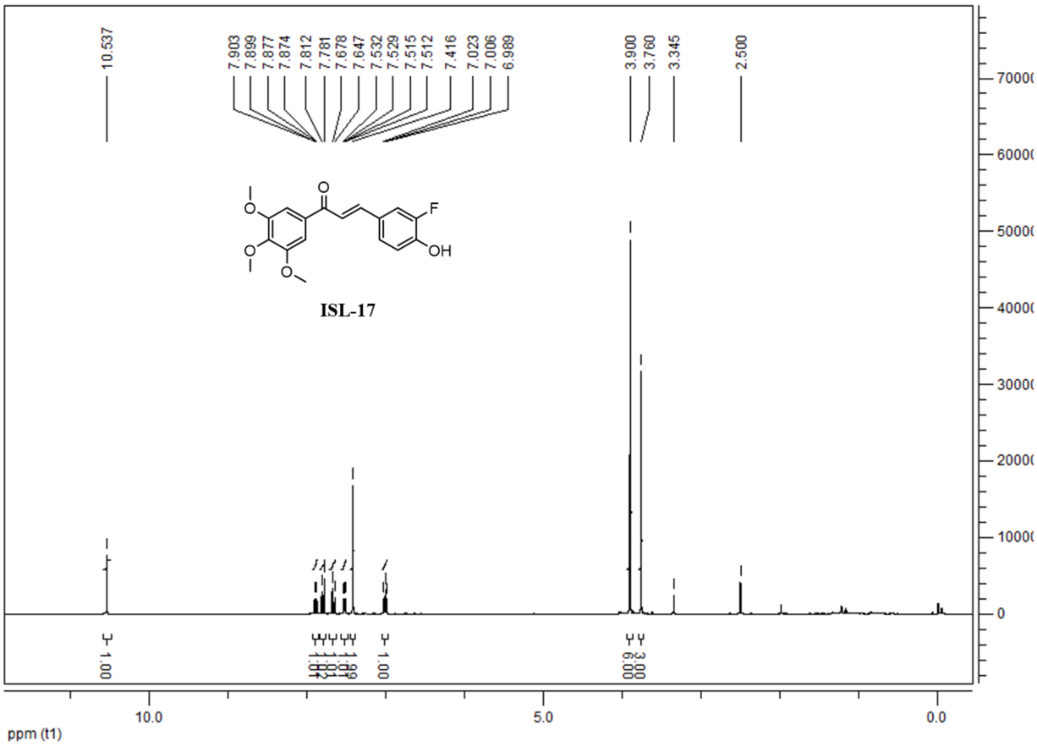

Figure S33. The ^{1}H NMR spectra of compound **ISL-17** (DMSO-d_6, Bruker AVANVEIII 400)

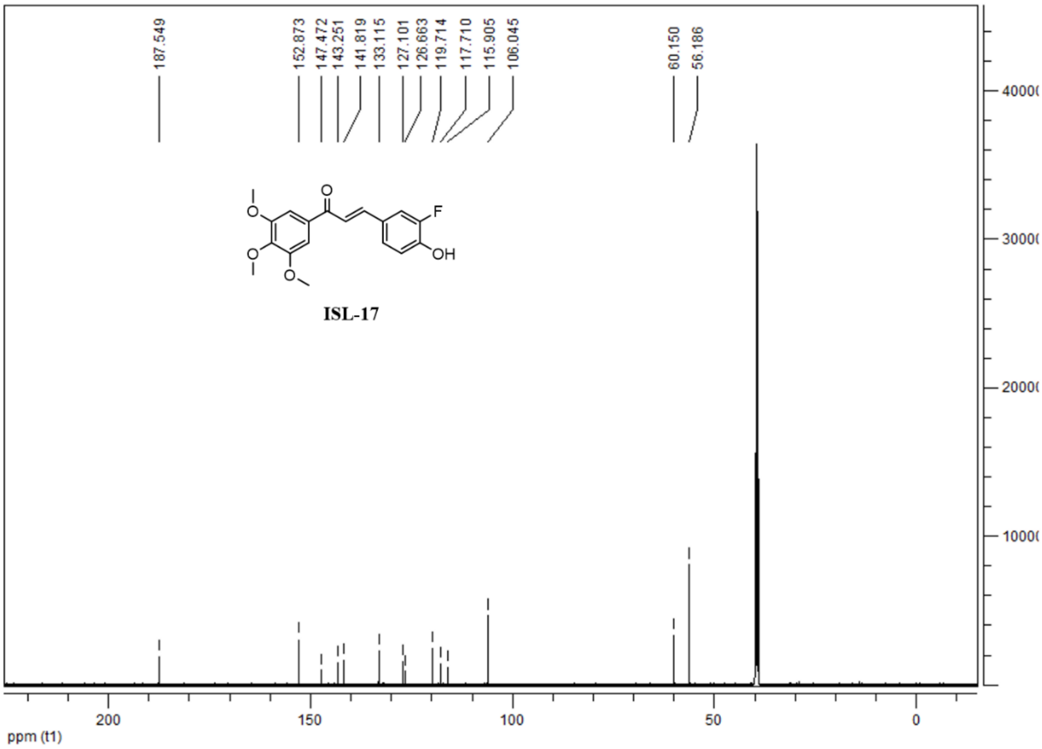

Figure S34. The ^{13}C NMR spectra of compound **ISL-17** (DMSO-d_6, Bruker AVANVEIII 400)

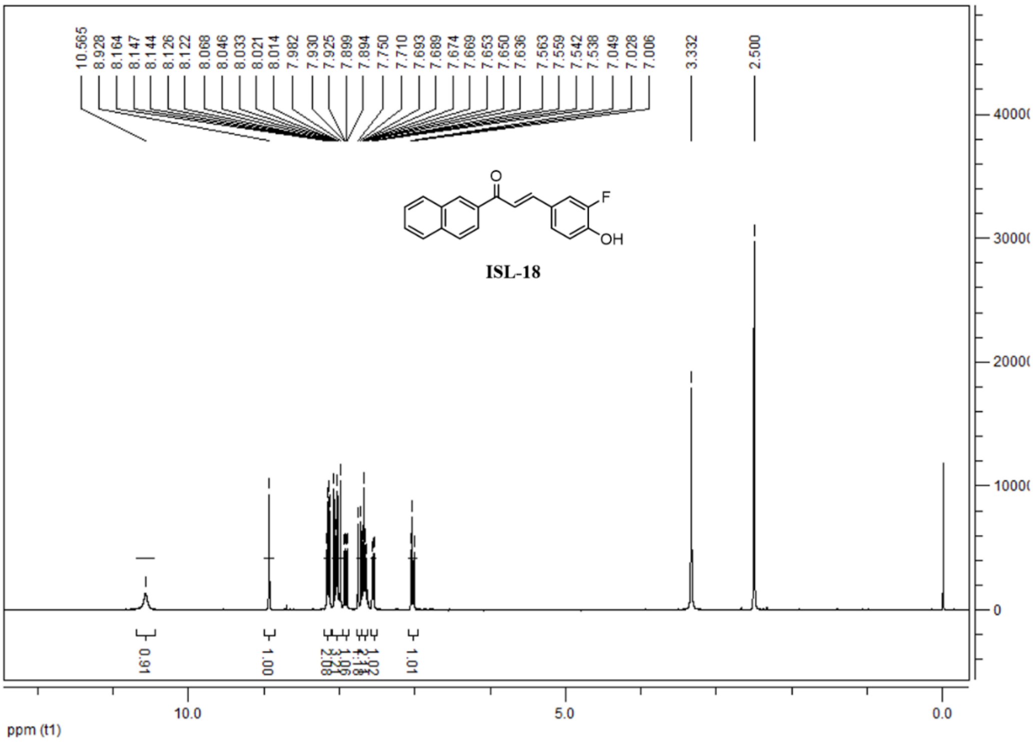

Figure S35. The ^{1}H NMR spectra of compound **ISL-18** (DMSO-d_6, Bruker AVANVEIII 400)

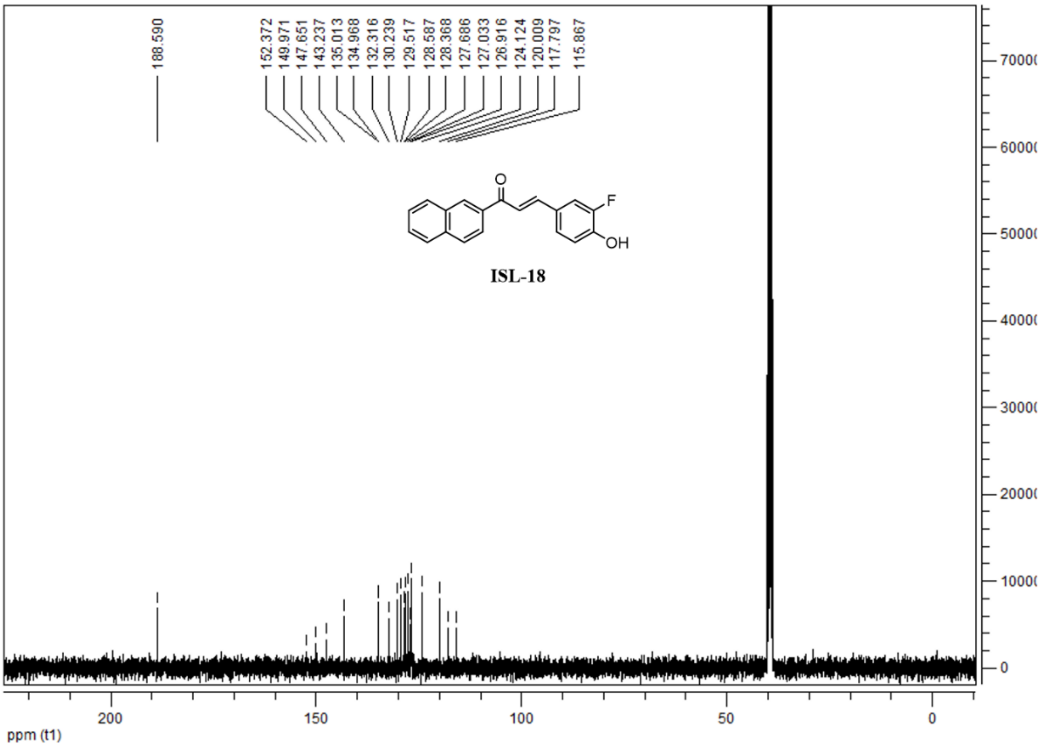

Figure S36. The ^{13}C NMR spectra of compound **ISL-18** (DMSO-d_6, Bruker AVANVEIII 400)

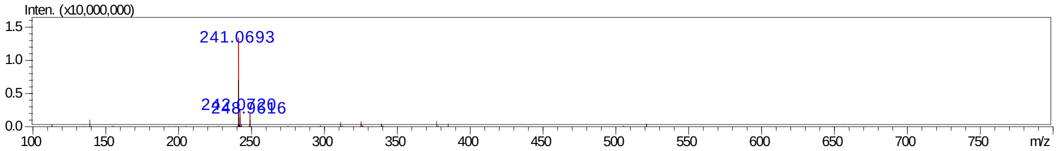

Figure S37. The High Resolution Mass Spectrometry (HRMS) spectra of compound **ISL-1** (HRMS (ESI, *m/z*) calcd. for $C_{15}H_{11}FO_2$ $(M-H)^-$ 241.0670, found 241.0693).

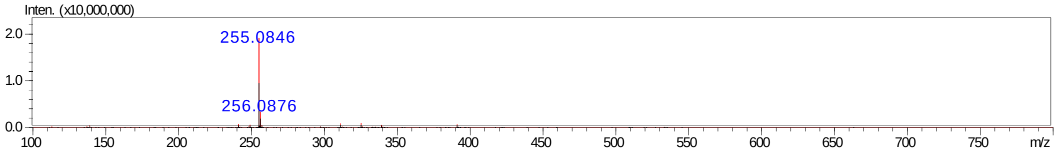

Figure S38. The HRMS spectra of compound **ISL-2** (HRMS (ESI, *m/z*) calcd. for $C_{16}H_{13}FO_2$ $(M-H)^-$ 255.0827, found 255.0846).

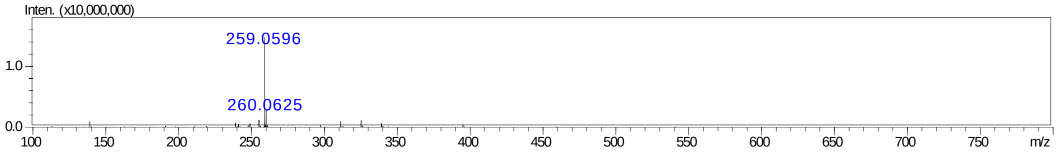

Figure S39. The HRMS spectra of compound **ISL-3** (HRMS (ESI, *m/z*) calcd. for $C_{15}H_{10}F_2O_2$ $(M-H)^-$ 259.0576, found 259.0596).

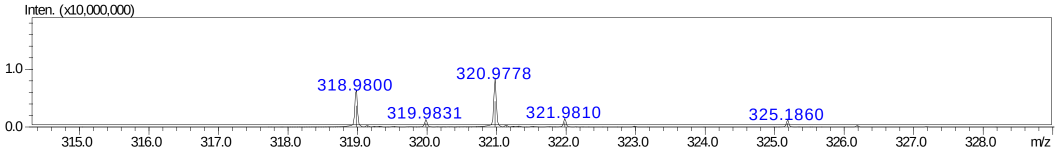

Figure S40. The HRMS spectra of compound **ISL-4 (**HRMS (ESI, *m/z*) calcd. for $C_{15}H_{10}BrFO_2$ $(M-H)^-$ 318.9775, found 318.9800).

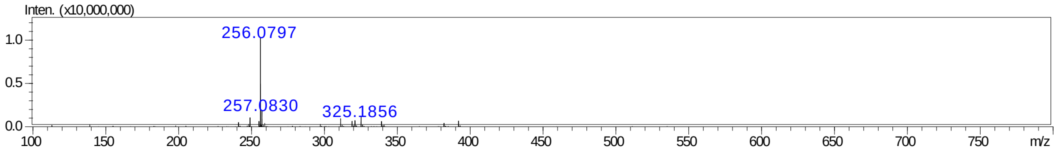

Figure S41. The HRMS spectra of compound **ISL-5** (HRMS (ESI, *m/z*) calcd. for $C_{15}H_{12}FNO_2$ $(M-H)^-$ 256.0779, found 256.0797).

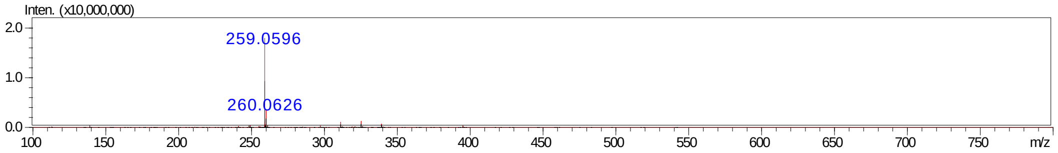

Figure S42. The HRMS spectra of compound **ISL-6** (HRMS (ESI, *m/z*) calcd. for $C_{15}H_{10}F_2O_2$ $(M-H)^-$ 259.0576, found 259.0596).

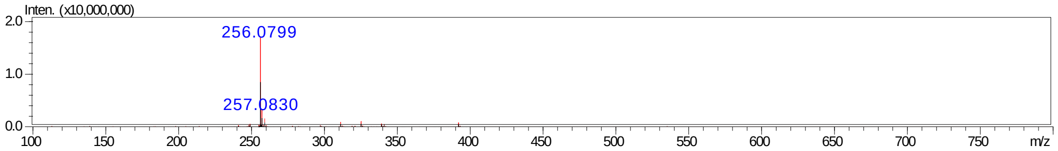

Figure S43. The HRMS spectra of compound **ISL-7** (HRMS (ESI, *m/z*) calcd. for $C_{15}H_{12}FNO_2$ $(M\text{-}H)^-$ 256.0779, found 256.0799).

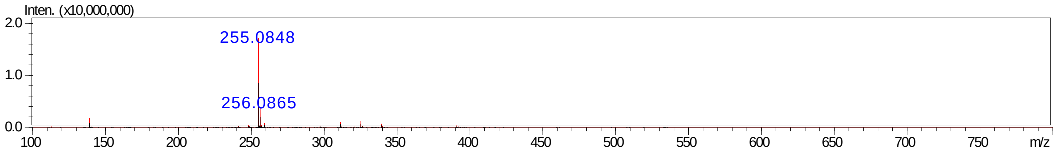

Figure S44. The HRMS spectra of compound **ISL-8** (HRMS (ESI, *m/z*) calcd. for $C_{16}H_{13}FO_2$ $(M\text{-}H)^-$ 255.0827, found 255.0848).

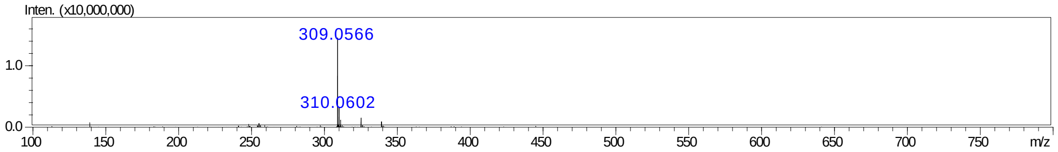

Figure S45. The HRMS spectra of compound **ISL-9** (HRMS (ESI, *m/z*) calcd. for $C_{16}H_{10}F_4O_2$ $(M\text{-}H)^-$ 309.0544, found 309.0566).

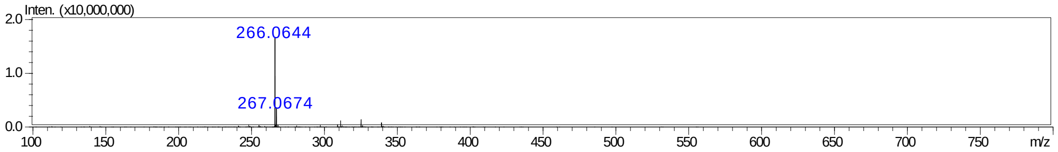

Figure S46. The HRMS spectra of compound **ISL-10** (HRMS (ESI, *m/z*) calcd. for $C_{16}H_{10}FNO_2$ $(M\text{-}H)^-$ 266.0623, found 266.0644).

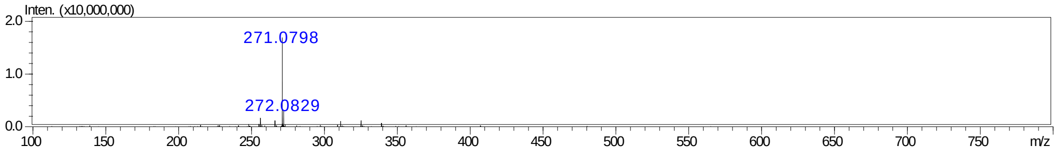

Figure S47. The HRMS spectra of compound **ISL-11** (HRMS (ESI, *m/z*) calcd. for $C_{16}H_{13}FO_3$ $(M\text{-}H)^-$ 271.0776, found 271.0798).

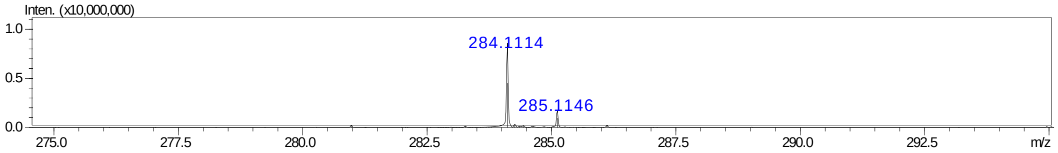

Figure S48. The HRMS spectra of compound **ISL-12** (HRMS (ESI, *m/z*) calcd. for $C_{17}H_{16}FNO_2$ $(M\text{-}H)^-$ 284.1092, found 284.1114).

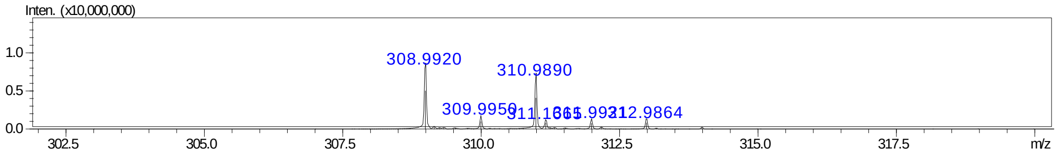

Figure S49. The HRMS spectra of compound **ISL-13** (HRMS (ESI, *m/z*) calcd. for $C_{15}H_9Cl_2FO_2$ $(M-H)^-$ 308.9891, found 308.9920).

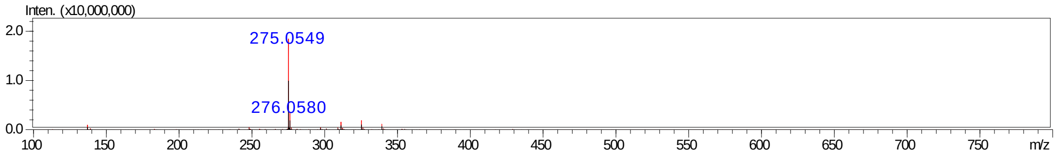

Figure S50. The HRMS spectra of compound **ISL-14** (HRMS (ESI, *m/z*) calcd. for $C_{15}H_{10}F_2O_3$ $(M-H)^-$ 275.0525, found 275.0549).

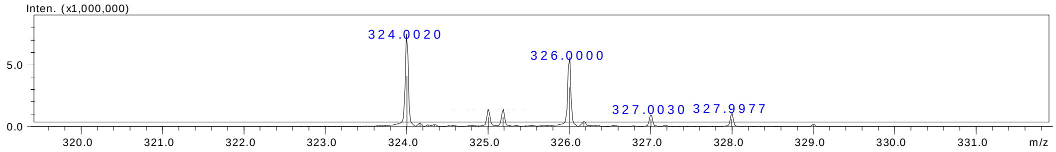

Figure S51. The HRMS spectra of compound **ISL-15** (HRMS (ESI, *m/z*) calcd. for $C_{15}H_{10}Cl_2FNO_2$ $(M-H)^-$ 324.0000, found 324.0020).

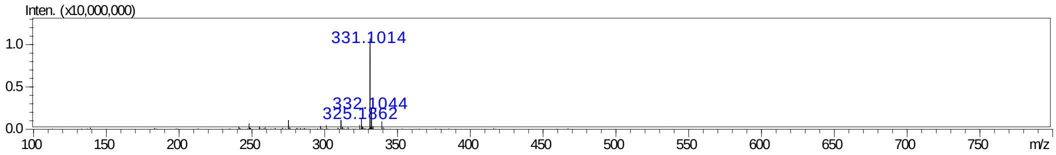

Figure S52. The HRMS spectra of compound **ISL-16** (HRMS (ESI, *m/z*) calcd. for $C_{18}H_{17}FO_5$ $(M-H)^-$ 331.0987, found 331.1014).

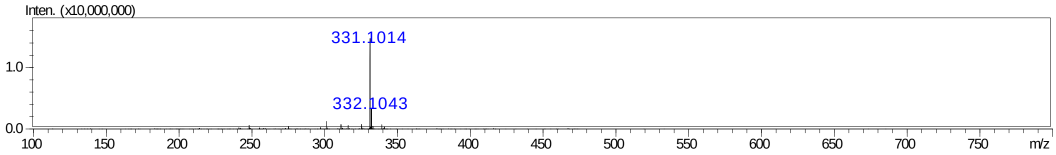

Figure S53. The HRMS spectra of compound **ISL-17** (HRMS (ESI, *m/z*) calcd. for $C_{18}H_{17}FO_5$ $(M-H)^-$ 331.0987, found 331.1014).

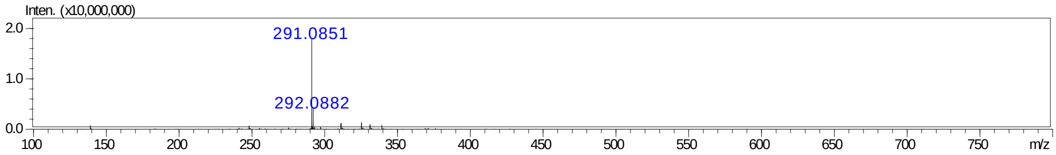

Figure S54. The HRMS spectra of compound **ISL-18** (HRMS (ESI, *m/z*) calcd. for $C_{19}H_{13}FO_2$ $(M-H)^-$ 291.0827, found 291.0851).
